# Poor Concordance of Floxed Sequence Recombination in Single Neural Stem Cells: Implications for Cell Autonomous Studies

**DOI:** 10.1523/ENEURO.0470-19.2020

**Published:** 2020-03-13

**Authors:** Tyler Joseph Dause, Elizabeth Diana Kirby

**Affiliations:** 1 Deptartment of Psychology, The Ohio State University, Columbus, OH 43210; 2 Deptartment of Neuroscience, The Ohio State University, Columbus, OH 43210; 3 The Chronic Brain Injury Program, The Ohio State University, Columbus, OH 43210

**Keywords:** adult neurogenesis, cell autonomous, CreER^T2^ recombinase, hippocampus, neural stem cells, subventricular zone

## Abstract

To manipulate target gene function in specific adult cell populations, tamoxifen (TAM)-dependent CreER^T2^ is widely used to drive inducible, site-specific recombination of loxP flanked sequences. In studies of cell autonomous target gene function, it is common practice to combine these CreER^T2^-lox systems with a ubiquitously expressed stop-floxed fluorescent reporter gene to identify single cells supposedly undergoing target gene recombination. Here, we studied the reliability of using Cre-induced recombination of one gene to predict recombination in another gene at the single-cell level in adult hippocampal neural stem and progenitor cells (NSPCs). Using both probabilistic predictions in a generic experimental paradigm, as well as a mouse model with two separate stop-floxed reporters plus a Nestin promoter-driven CreER^T2^, we found that, in individual cells, recombination of one gene was a poor predictor of recombination in another. This poor concordance in floxed sequence recombination across genes suggests that use of stop-floxed reporters to investigate cell autonomous gene function may not be universally reliable and could lead to false conclusions.

## Visual Abstract

**Figure:**
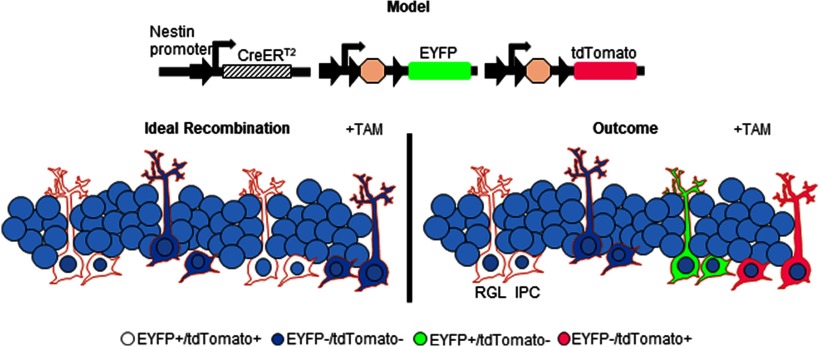

## Significance Statement

We investigate the reliability of a widely used transgenic mouse model in studies of adult neural stem and progenitor cells (NSPCs). Ligand-dependent Cre recombinases, such as the CreER^T2^ model, are a fundamental tool for inducible gene modification used to investigate gene function in many cell populations. It is common practice to combine NSPC-specific CreER^T2^-lox systems with a ubiquitously expressed stop-floxed fluorescent reporter gene to identify single cells undergoing target gene recombination in studies of cell autonomous gene function. Our probabilistic predictions and experimental data suggest that use of stop-floxed reporters to investigate cell autonomous gene function in NSPCs may lead to false conclusions because recombination in separate genes can show poor concordance in individual cells.

## Introduction

In the adult mammalian brain, there are two primary neurogenic niches where neural stem and progenitor cells (NSPCs) proliferate throughout life: the subventricular zone (SVZ) and the dentate gyrus (DG) of the hippocampus (Gage, 2000). NSPCs in these regions create new neurons during adulthood, which incorporate into the preexisting circuitry in the olfactory bulb or DG, respectively, a process known as neurogenesis (Zhao et al., 2008). As evidence has mounted for the conservation of adult neurogenesis across species (Kempermann, 2015), understanding the functional properties of adult neurogenesis, as well as the molecular mechanisms that regulate it, has emerged as a major focus of research.

A common and powerful approach to dissecting molecular mediators of complex cellular processes like neurogenesis relies on gene ablation or overexpression to simulate gain or loss of function of target proteins. Genetic manipulation of adult neurogenesis requires cell-specific, inducible models that affect adult NSPCs selectively without influencing developmental counterparts. Ligand-dependent Cre recombinases, such as the tamoxifen (TAM)-dependent CreER^T2^, are widely-used for driving such inducible, site-specific recombination of loxP flanked sequences in adult NSPCs (Feil et al., 2009; Semerci and Maletic-Savatic, 2016). Several CreER^T2^ mouse lines are currently available which use NSPC-specific promoters to drive Cre expression and therefore TAM-induced recombination in adult NSPCs (Sun et al., 2014; Yang et al., 2015; Semerci and Maletic-Savatic, 2016). In many studies, NSPC-targeted CreER^T2^-lox systems are combined with a ubiquitously-expressed stop-floxed fluorescent reporter gene to confirm recombination in NSPCs (Sun et al., 2014; Kirby et al., 2015; Tannenholz et al., 2016). It is also common to use these fluorescent proteins more cell-specifically to investigate cell autonomous effects of recombination in the experimental gene of interest (Zimmermann et al., 2018; Zhou et al., 2018; Franz et al., 2019; Zhang et al., 2019). These cell autonomous experimental paradigms suppose that target gene and fluorescent reporter gene recombination occur in the same cell with high probability, allowing investigators to identify cell autonomous effects by comparing fluorescent and non-fluorescent cells.

To our knowledge, the assumption that fluorescent reporter expression reliably equates to recombination in a separate gene in the same cell has yet to be scrutinized. Within a cell, each recombination event is independent, and, with a transiently-activated Cre such as CreER^T2^, divergence of recombination status across loci seems possible. Here, we examine the reliability of using Cre-induced recombination of one gene to predict recombination in another gene at the single-cell level in adult NSPCs. Using one common inducible Cre-driver mouse line and two, widely-used stop-floxed fluorescent reporter lines, we found that Cre-induced expression of one fluorescent reporter did not reliably predict expression of the other within a single cell *in vivo*. Given our results, we suggest that stop-floxed fluorescent reporters may misrepresent cell autonomous effects of gene recombination at a cell-specific level and that it may not be prudent to assume any specific pairing of reporter and target genes shows high recombination concordance in single cells without other, confirmatory data.

## Materials and Methods

### Mice

NestinCreER^T2^ mice (Jackson #016261) were crossed with two conditional reporter lines: Rosa-stop-floxed-EYFP (Srinivas et al., 2001; Jackson #006148) and Rosa-CAG-stop-floxed-tdTomato (Madisen et al., 2010: Ai9; Jackson #007909). Mice were bred and maintained in The Ohio State University Psychology building mouse vivarium in standard ventilated cages on a 12/12 h light/dark cycle (lights on 6:30 A.M.), with *ad libitum* access to food and water. Male and female mice were eight to nine weeks old at the time of the experiment and housed in groups of two to four. All animal use was in accordance with institutional guidelines approved by The Ohio State University Institutional Animal Care and Use Committee.

### TAM administration

TAM was dissolved in sterile sunflower oil at 20 mg/ml, overnight with agitation at 37°C. TAM solution was stored at +4°C for up to one week. TAM (or oil vehicle) was injected (180 mg/kg/d, i.p.) for 3 or 5 d.

### Immunofluorescent staining/antibodies

After 2 d (3D short/5D mice) or 4 d (3D long mice), mice were anesthetized with an 87.5 mg/kg ketamine, 12.5 mg/kg xylazine mixture and then transcardially perfused with ice-cold 0.1 M PBS. Harvested brains were fixed in 4% paraformaldehyde in 0.1 M phosphate buffer overnight at 4°C. After equilibration in 30% sucrose in PBS, 40-μm coronal brain sections were obtained in one in 12 series on a freezing microtome (Leica), and stored in cryoprotectant at −20°C until use. Brain sections were rinsed with PBS three times then incubated in a blocking solution containing 1% normal donkey serum (Jackson ImmunoResearch) and 0.3% Triton X-100 (Acros) in PBS. Sections were then incubated in primary antibody (Table 1) diluted in blocking solution overnight at 4°C with rotation. The following day, after three rinses in PBS, cells were incubated in secondary antibodies (Table 1) diluted 1:500 in blocking solution for 2 h with rotation. The DG of the hippocampus was imaged in 15-μm z-stacks at 20× magnification using a Zeiss Axio Observer Z.1 with apotome digital imaging system and Axiocam 506 monochrome camera (Zeiss).

**Table 1:** Primary and secondary antibodies

Primary antibody	Vendor/ product no.	Dilution	Secondary	Vendor/product no.	Dilution
Rabbit anti-mCherry	Abcam ab167453	1:500	Donkey anti-Rabbit IgG (H+L) highly cross-adsorbed secondary antibody, AlexaFluor 555	ThermoFisher Scientific A-31572	1:500
Hoechst	Fisher 33342	1:2000	N/A	N/A	N/A
Goat Anti-GFP antibody	Abcam Ab6673	1:1000	Donkey anti-Goat IgG (H+L) cross-adsorbed secondary antibody, AlexaFluor 488	Fisher A-11055	1:500
Mouse Anti-glial fibrillary acidic protein, clone GA5 (GFAP)	EMD Millipore MAB360	1:1000	Donkey anti-mouse IgG (H+L) highly cross-adsorbed secondary antibody, Alexa Fluor 647	Fisher A-31571	1:500
Rat anti-Ki-67 monoclonal antibody	Invitrogen 14-5698-82	1:500	AlexaFluor 647 AffiniPure donkey anti-Rat IgG (H+L) (712-605-153)	Jackson ImmunoResearch 712-605-153	1:500

### Automated image processing

Colocalization of immunofluorescent signal was analyzed with just another colocalization plugin (JACoP) software on ImageJ (Bolte and Cordelières, 2006). First, z-stacks from each DG were separated into individual 1-μm-thick images, 1 image per fluorescent channel. Images were thresholded then overlapped using anatomic features. The JACoP plugin then re-stacked the image files and analyzed overlap of EYFP and tdTomato. These data were used to determine EYFP and tdTomato percent area, the proportion of EYFP overlapping tdTomato and the proportion of tdTomato overlapping EYFP.

### NSPC identification and manual cell counts

Radial glia-like NSCs (RGLs) were identified by their GFAP+ radial processes extending from the subgranular zone (SGZ) into the molecular layer, while cells with Ki-67+ cell bodies in the SGZ layer were identified as intermediate progenitor cells (IPCs). All NSPCs were counted in each section. RGLs and IPCs were manually identified in 1-μm z-stack images using ImageJ then assessed for EYFP or tdTomato coexpression as described. Density of RGLs and IPCs was determined as the number of cells per area in the DG/SGZ. The SGZ was defined as the zone spanning two cell body widths between the dense granular cell layer and the hilus.

### Statistical analysis

Comparisons of more than two groups were performed using one-way or two-way ANOVAs followed by Tukey’s multiple comparisons. Correlations were performed using Pearson’s correlation. One-sample *t* tests were used to compare difference from a theoretical value of 100%. All analyses were performed using Prism (v8.0; GraphPad Software), and *p* < 0.05 was considered significant (Table 2; Extended Data Tables 2-1, 2-2).

**Table 2:** Statistics table

EYFP and tdTomato DG percentage area comparison							
	TAM administration	*n*	Test	Comparison	Statistic	*P*	Significant
2*E*	3D Short	3	Two-way ANOVA	TAM × reporter	*F*_(2,6)_ = 8.880	0.0161	Yes*
2*E*	3D Long	3		TAM	*F*_(2,6)_ = 11.32	0.0092	Yes**
2*E*	5D	3		Reporter	*F*_(1,6)_ = 14.01	0.0096	Yes**
				Subject	*F*_(6,6)_ = 12.52	0.0036	Yes**
			
				EYFP		tdTomato	
	*Post hoc*		Comparisons	Adjusted *P*	Significant	Adjusted *P*	Significant
	Tukey’s multiple comparisons		3D short vs 3D long	≥0.9999	No	0.9976	No
			3D short vs 5D	0.0009	Yes***	0.0274	Yes*
			3D long vs 5D	0.0009	Yes***	0.0308	Yes*
			
EYFP and tdTomato DG percentage area correlation							
Figure	TAM administration	*n*	Test	Statistic	*P*	Significant	
2*F*	All	9	Pearson's Correlation	*r*_(8)_ = 0.9157	0.0005	Yes***	
			
EYFP+ percentage area colocalization with tdTomato+ area							
	TAM administration	*n*	Test	Statistic	*P*	Significant	
2*H*	3D Short	3	One-sample *t* test w/ comparison to 100%	*t*_(2)_ = 35.87	0.0008	Yes***	
2*H*	3D Long	3		*t*_(2)_ = 13.15	0.0057	Yes**	
2*H*	5D	3		*t*_(2)_ = 15.36	0.0042	Yes**	
			
tdTomato+ percentage area colocalization with EYFP+ area							
Figure	TAM administration	*n*	Test	Statistic	*P*	Significant	
2*I*	3D Short	3	One-sample *t* test w/ comparison to 100%	*t*_(2)_ = 119.7	0.0001	Yes****	
2*I*	3D Long	3		*t*_(2)_ = 10.40	0.0091	Yes**	
2*I*	5D	3		*t*_(2)_ = 11.27	0.0078	Yes**	
			
DG percentage of NSPCs with True+ Signal							
Figure	TAM administration	*n*	Test	Comparison	Statistic	*P*	Significant
4*B*	3D Short	3	Two-way ANOVA	True signal × Tam	*F*_(2,6)_ = 6.084	0.0360	Yes*
4*B*	3D Long	3		True signal	*F*_(1,6)_ = 3.215	0.1231	No
4*B*	5D	3		TAM	*F*_(2,6)_ = 3.201	0.1132	No
				Subject	*F*_(6,6)_ = 0.02808	0.9998	No

			*Post hoc*	Comparisons	Adjusted *P*	Significant	
			Tukey’s multiple comparisons	3D short vs 3D long	0.8875	No	
				3D short vs 5D	0.0225	Yes*	
				3D long vs 5D	0.0516	No	
DG percentage of NSPCs with True−Signal							
Figure	TAM administration	*n*	Test	Comparison	Statistic	*P*	Significant
4*B*	3D Short	3	Two-way ANOVA	True−Signal × TAM	*F*_(2,6)_ = 6.084	0.0360	Yes*
4*B*	3D Long	3		True−Signal	*F*_(1,6)_ = 3.215	0.1231	No
4*B*	5D	3		TAM	*F*_(2,6)_ = 3.201	0.1132	No
				Subject	*F*_(6,6)_ = 0.02808	0.9998	No
			
			*Post hoc*	Comparisons	Adjusted *P*	Significant	
			Tukey’s multiple comparisons	3D short vs 3D long	0.9389	No	
				3D short vs 5D	0.0328	Yes*	
				3D long vs 5D	0.0179	Yes*	
			
DG percentage of NSPCs with True ± Signal							
Figure	TAM administration	*n*	Test	Statistic	*P*	Significant	
4*C*	3D Short	3	One-way ANOVA	*F*_(2,6)_ = 3.201	0.1132	No	
4*C*	3D Long	3		*F*_(2,6)_ = 3.201	0.1132	No	
4*C*	5D	3		*F*_(2,6)_ = 3.201	0.1132	No	

*p < 0.05, **p < 0.01,***p < 0.001, ****p < 0.0001.

10.1523/ENEURO.0470-19.2020.t2-1 Supplementary Extended Data Table 2-1 Main Figures Raw Data. Download Table 2-1, DOCX file.

10.1523/ENEURO.0470-19.2020.t2-2 Supplementary Extended Data Table 2-2 Extended Data Figures Raw Data and Statistics. Download Table 2-2, DOCX file.

## Results

**Figure 1.**
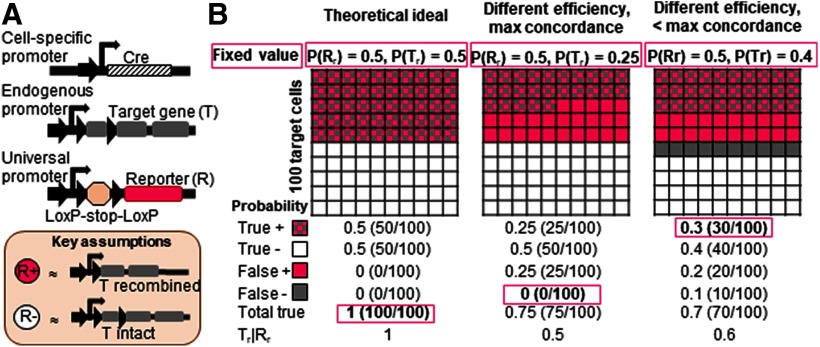
Theoretical probabilistic models reveal potential for error in a common paradigm for testing cell autonomous gene function. ***A***, Cell autonomous function of genes is frequently investigated using a cell-specific Cre-loxP system where expression of reporter protein from a stop-floxed reporter construct is assumed to reliably indicate target gene recombination within a single cell. Here, a generic schematic of the genetics used and assumptions employed in this common paradigm are shown. ***B***, The probabilities of true and false reporter signals are shown for three hypothetical scenarios with 100 target cells for visualization. All three scenarios assume an experiment where gene recombination occurs only in the presence of Cre, reporter protein expression is observed in 50% of target cells [P(R_r_) = 0.5], and reporter recombination is greater than or equal to target gene recombination [P(R_r_) ≥ P(T_r_)]. The numbers in red boxes represent given values that are then used to determine the remaining probabilities. The “theoretical ideal” represents an ideal scenario where reporter gene recombination and target gene recombination overlap perfectly. The “different efficiency, max concordance” scenario shows a case where target gene recombines with less efficiency than the reporter, but shows the maximum possible concordance with reporter recombination given that constraint. The “different efficiency, < max concordance” scenario shows a case where target gene recombination is mildly less efficient than reporter gene recombination and concordance is moderately suboptimal, with 75% of target recombined cells also showing reporter recombination. In the hypothetical deviations from ideal, the probability of reporter expression accurately predicting target gene recombination [P(T_r_|R_r_)] is 0.5 and 0.6, respectively. These examples demonstrate that even mild deviation from ideal can introduce possible substantial error in using reporter gene recombination as an indicator of target gene recombination in the same cell. See also Extended Data Figure 1-1. *Figure Contributions*: Elizabeth Diana Kirby developed theoretical models and made figures.

First, we explored the theoretical accuracy of using Cre-induced recombination of one gene to predict recombination in another gene. We chose to model an experimental paradigm where recombination of a stop-floxed fluorescent reporter (Gene R) is used as a marker of recombination in a target gene (Gene T) to make conclusions about the cell autonomous effect of target gene recombination (Fig. 1*A*). This design assumes that detection of reporter protein indicates target gene recombination with high probability (a true positive signal) and absence of reporter protein indicates lack of target gene recombination with high probability (a true negative signal; Fig. 1*B*).

10.1523/ENEURO.0470-19.2020.f1-1 Extended Data Figure 1-1 Equation derivation for true and false signal probabilities. ***A***, Assuming reporter recombination is greater than or equal to target gene recombination [P(R_r_) ≥ P(T_r_)], probability of total true signal is derived using standard conditional and unconditional probability formulas. ***B–D***, Application of the equations in ***A*** to the scenarios described in Figure 1*B* are shown in full detail. *Figure Contributions*: Elizabeth Diana Kirby developed theoretical models. Download Figure 1-1, TIF file.

To explore the consequences of deviations from the ideal of high true signal fraction, we performed probabilistic calculations of true signals based on several simplifying assumptions. First, we assumed recombination of gene T to be an all-or-none outcome (i.e., no possibility of heterozygosity). Next, we assumed that recombination does not occur in either gene when Cre is not expressed (i.e., minimal ectopic recombination). In many transgenic models, this assumption has been shown to hold true (Imayoshi et al., 2006; Kim et al., 2011; Yang et al., 2015). Third, we assumed that the probability of reporter recombination is larger than or approximately equal to the probability of target gene recombination [P(R_r_) ≥ P(T_r_)]. This assumption is based on the likely selection of stop-floxed fluorescent reporter constructs that recombine with high efficiency. Given these constraints, P(total true) = P(true +) + P(true –) = 1 + 2 * P(R_r_) * P(T_r_|R_r_) – P(R_r_) – P(T_r_) (equation derivation in Extended Data Fig. 1-1*A*).

In most experimental paradigms, P(R_r_) is known based on immunohistochemical quantification of percent of target cells expressing the reporter. For our estimates, we assumed an experimentally-feasible P(R_r_) = 0.5 (i.e.,50% of target cells showing recombination-dependent reporter expression). In the ideal outcome for this experiment, where total true signal is 100%, 50/100 cells would show simultaneous reporter expression and target gene recombination (Fig. 1*B*; Extended Data Fig. 1-1*B*, “theoretical ideal”). However, while reporter gene recombination is frequently used as an estimate of target gene recombination, there is ample evidence that efficiency of Cre-loxP recombination varies from gene to gene (Long and Rossi, 2009; Sun et al., 2014; Gray et al., 2017). We therefore examined the effect of setting P(R_r_) = 0.5 but P(T_r_) = 0.25. Using the best possible scenario where target gene recombination occurs only in reporter gene recombined cells [i.e., P(false –) = 0], we found that P(T_r_|R_r_) = 0.5, meaning that identifying a cell as reporter positive only yields a 50% chance that the cell is also target gene recombined (Fig. 1*B*; Extended Data Fig. 1-1*C*, “different efficiency, max concordance”).

We next estimated the effect of a less dramatic difference between P(R_r_) and P(T_r_) but when mixed with moderately suboptimal recombination concordance between the two genes within one cell. Using P(T_r_) = 0.4 and assuming that 75% of target recombined cells are also reporter recombined [i.e., P(T_r_) * 0.75 = P(true +)], we found that observing a reporter-expressing cell only yielded a 60% chance that that cell was also target gene recombined [i.e., P(T_r_|R_r_) = 0.6]. Observing a reporter-negative cell in this case yielded a 10% chance that that cell was target gene recombined [P(false –) = 0.1; Fig. 1*B*; Extended Data Fig. 1-1*D*, “different efficiency, < max concordance”]. These findings suggest that seemingly mild variation in recombination efficiency and suboptimal concordance rates for gene recombination could introduce substantial error in the method of using reporter expression as a marker of target gene recombination within a single cell.

**Figure 2.**
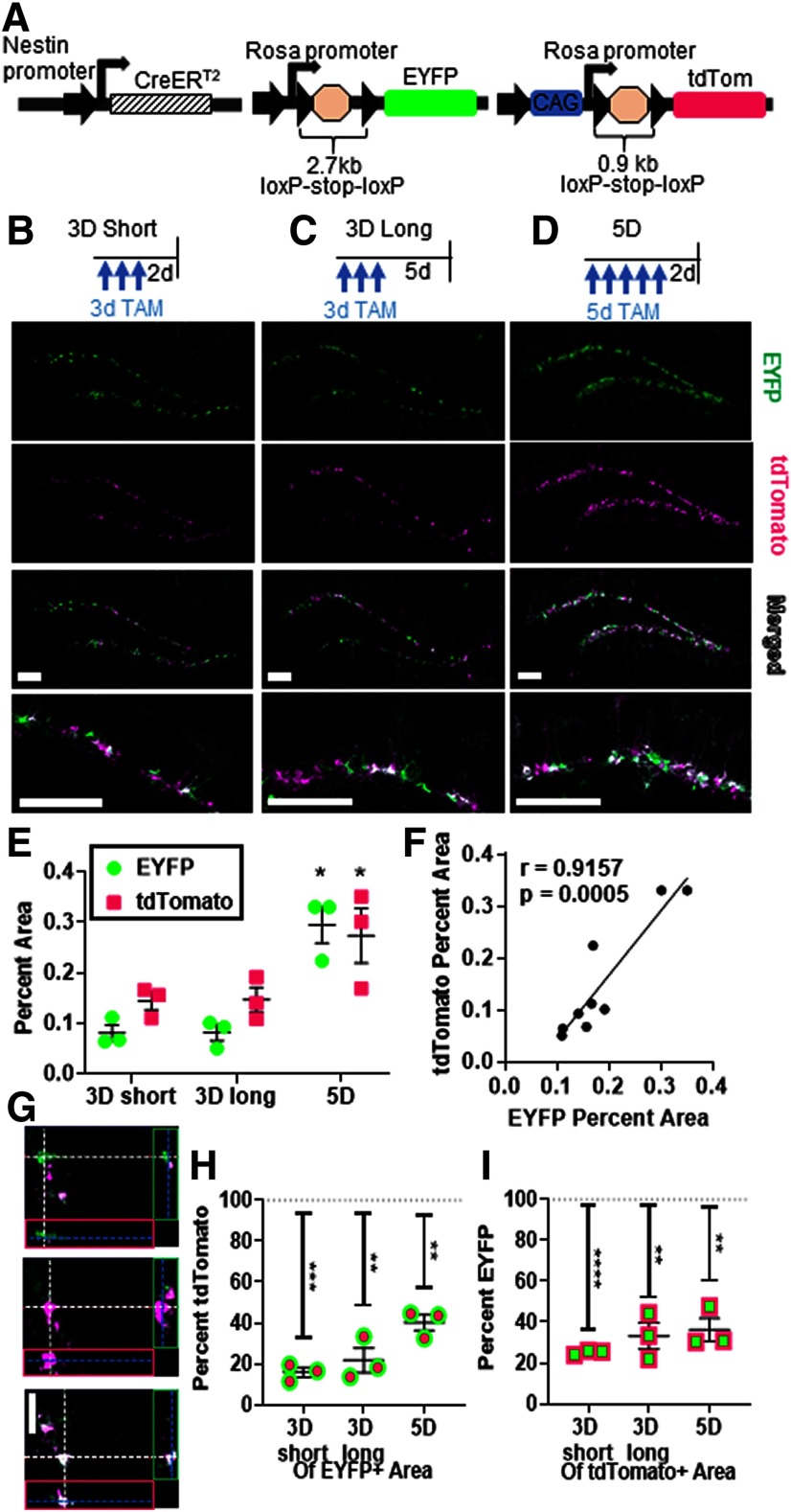
Recombination-dependent fluorescent reporter gene expression is correlated over the whole SGZ, but frequently fails to co-localize. ***A***, A schematic of the transgenic mouse model employed in our experiments where we combined NestinCreER^T2^ mice Rosa-stop-floxed-EYFP and Rosa-CAG-stop-floxed-tdTomato mice. Mice were then submitted to three TAM administration conditions, 3D short (***B***), 3D long (***C***), and 5D (***D***; top, representative timeline of TAM injections and recovery; bottom, immunostaining of EYFP and tdTomato in SGZ; scale bars: 100 μm). ***E***, EYFP+ and tdTomato+ DG percent area was compared by two-way ANOVA in 3D short, 3D long, and 5D mice. Tukey’s *post hoc* comparison within reporter type; *p < 0.05, **p < 0.01, ***p < 0.001 versus 5D. ***F***, EYFP+ and tdTomato+ DG percent area were correlated in all mice. *R*,p Pearson’s correlation. ***G***, Representative orthogonal images: immunostaining and imaging of EYFP+, tdTomato+, and EYFP+tdTomato+ cells in the SGZ. Scale bar: 20 μm. ***H***, EYFP+ colocalization in tdTomato+ area (***H***) and tdTomato+ colocalization in EYFP+ area (***I***) were compared with theoretical 100% colocalization; *n* = 3 mice per group. Data are shown as mean ± SEM; *p < 0.05, **p < 0.01, ***p < 0.001, ****p < 0.0001. *Figure Contributions*: Tyler Joseph Dause ran experiments and analyzed data. Tyler Joseph Dause and Elizabeth Diana Kirby made figure.

To test the accuracy of using Cre-induced recombination of one gene to predict recombination of another gene in an in vivo experimental paradigm, we combined NestinCreER^T2^ mice with two commonly-used conditional reporter lines: Rosa-stop-floxed-EYFP (Srinivas et al., 2001) and Rosa-CAG-stop-floxed-tdTomato (Madisen et al., 2010; Fig. 2*A*). NestinCreER^T2^ mice express a TAM-sensitive Cre recombinase that drives recombination of floxed sequences in Nestin-expressing NSPCs in the adult brain (Lagace et al., 2007). The floxed sequences used, Rosa-stop-floxed-EYFP and Rosa-CAG-stop-floxed-tdTomato genes, are both inserted in the Rosa locus, although the tdTomato construct also includes an additional CAG promoter that the EYFP construct does not (Fig. 2*A*; Srinivas et al., 2001; Madisen et al., 2010).

First, to verify the TAM-dependency of reporter gene recombination in this model, adult NestinCreER^T2^;Rosa(EYFP/tdTomato) mice were injected with oil (vehicle) or TAM once per day for 5 d. This TAM dosing schedule is widely-used and leads to efficient and specific recombination of floxed genetic sequences in NSPCs in adult NestinCreER^T2^ mice (Sun et al., 2014). As expected, oil-injected mice exhibited negligible ectopic recombination of either reporter gene (Extended Data Fig. 2-1*A*) while TAM administration induced robust fluorescent reporter expression in DG NSPCs (Extended Data Fig. 2-1*B*). These findings indicate that recombination of both reporter genes is tightly dependent on TAM, as found in previous studies (Imayoshi et al., 2006; Kim et al., 2011; Yang et al., 2015).

10.1523/ENEURO.0470-19.2020.f2-1 Extended Data Figure 2-1 Comparison of oil- and TAM-injected adult NestinCreER^T2^;Rosa(EYFP/tdTom) mice. ***A***, Immunostaining in the adult DG shows that oil administration does not stimulate expression of either reporter gene. ***B***, TAM administration induces robust recombination-dependent expression of both EYFP and tdTomato in the SGZ. Scale bars: 100 μm. *Figure Contributions*: Tyler Joseph Dause ran TAM experiments and tissue staining. Tyler Joseph Dause and Elizabeth Diana Kirby made figures. Download Figure 2-1, TIF file.

We next examined the population-level efficiencies of recombination in both fluorescent reporter genes to determine if recombination frequency in one gene is a general predictor of recombination in a separate gene. To create a range of recombination rates, NestinCreER^T2^;Rosa(EYFP/tdTom) mice were submitted to one of three different TAM administration and recovery protocols: 3 d of TAM with 3 d of recovery (3D short), 3 d of TAM with 5 d of recovery (3D long) and 5 d of TAM with 3 d of recovery (5D), the standard TAM regimen (Fig. 2*B–D*). Analysis of the percent area occupied by fluorescent reporter proteins in the DG confirmed that a range of recombination rates was achieved, with 5D TAM leading to the highest level of reporter expression (*F*_(2,6)_ = 11.32, *p* = 0.0092; two-way ANOVA; *p* < 0.05 Tukey’s; Fig. 2*E*). Across all three TAM groups, there was a strong and significant correlation between tdTomato and EYFP expression in the DG of TAM injected mice (*r*_(8)_ = 0.9157, *p* = 0.0005; Pearson’s correlation; Fig. 2*F*). These findings suggest that mice with high recombination of one reporter gene also have high recombination of the other and support the possibility that recombination of one gene might be predictive of recombination in a second gene.

To examine reporter coexpression within single cells, we quantified EYFP and tdTomato antibody-amplified fluorescence co-localization in 1-μm z-stack image series from adult DG. In these images, cells expressing EYFP alone appear green and cells expressing tdTomato alone appear magenta, while cells expressing both fluorescent reporters appear white (Fig. 2*G*). Qualitative assessment of reporter expression revealed representation of each of these three possible colocalization phenotypes (Fig. 2*G*). Using an automated co-localization tool to quantify reporter overlap, we found that EYFP+ percent area colocalization with tdTomato+ signal ranged from 15.84% in 3D short mice and 21.77% in 3D long mice to 36.71% in 5D mice, all of which were significantly less than 100% (3Ds: *t*_(2)_ = 35.87, *p* = 0.0008 3Dl: *t*_(2)_ = 13.15, *p* = 0.0057 5D: *t*_(2)_ = 15.36, *p* = 0.0042; one-sample *t* test comparison to 100%; Fig. 2*H*). The converse, tdTomato+ percent area of EYFP+ area, showed slightly higher colocalization frequencies: 3D short 25.14%, 3D long 33.22%, and 5D 36.30%, although all still significantly less than 100% (3Ds: *t*_(2)_ = 119.7, *p* = 0.0001 3Dl: *t*_(2)_ = 10.40, *p* = 0.0091 5D: *t*_(2)_ = 11.27, *p* = 0.0078; one-sample *t* test comparison to 100%; Fig. 2*I*). These results suggest that the fluorescent markers may not be coexpressed in the same NSPCs with high frequency.

**Figure 3.**
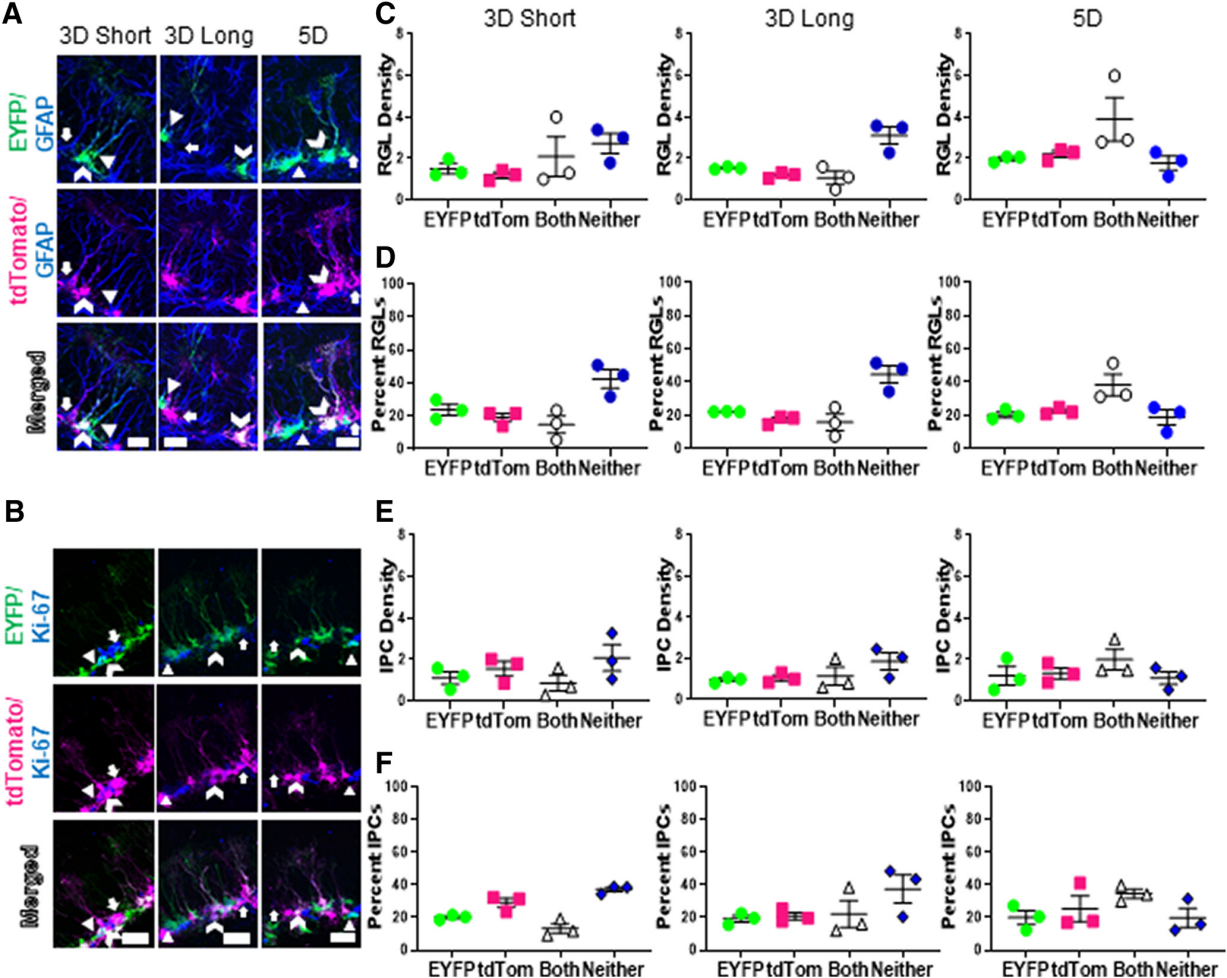
Recombination-dependent fluorescent reporter gene expression within RGLs and IPCs frequently fails to co-localize in the same cells. ***A***, SGZ RGLs were identified by immunostaining of EYFP, tdTomato, and GFAP. Scale bars: 20 μm. Arrow head = EYFP+/tdTomato– RGL. Arrow = EYFP–/tdTomato+ RGL. Chevron = EYFP+/tdTomato+ RGL. ***B***, SGZ IPCs were identified by immunostaining of EYFP, tdTomato and Ki-67. Scale bars: 20 μm. Arrow head = EYFP+/tdTomato– IPC. Arrow = EYFP–/tdTomato+ IPC. Chevron = EYFP+/tdTomato+ IPC. Density (***C***) and percentage (***D***) of SGZ GFAP+ RGLs coexpressing EYFP only(EYFP), tdTomato only(tdTom), EYFP+ and tdTomato+ (both), or neither are shown. Density (***E***) and percentage (***F***) of SGZ Ki67+ IPCs coexpressing EYFP, tdTom, both, or neither are shown; *n* = 3 mice per group. Data are shown as mean ± SEM. Density represented as cells per area, scale × 10^−4^. See also Extended Data Figure 3-1. *Figure Contributions*: Tyler Joseph Dause ran experiments and analyzed data. Tyler Joseph Dause and Elizabeth Diana Kirby made figure.

To quantify recombination frequencies within NSPC subpopulations in the SGZ, we used immunofluorescent labeling for phenotypic markers to identify reporter expression in RGLs and IPCs (Fig. 3*A*,*B*). RGLs were identified based on a cell body in the SGZ with a GFAP+ radial process extending through the granule cell layer (Lugert et al., 2010; Fig. 3*A*). IPCs were identified as Ki-67+ nuclei in the SGZ (Mandyam et al., 2007; Fig. 3*B*). Recombination frequency of each reporter gene individually was similar to that reported in previous studies using NestinCreER^T2^ mice (Extended Data Fig. 3-1*A–D*). In addition, similar to our findings using fluorescent reporter area, recombination rates of EYFP and tdTomato reporter genes were positively associated [*r*_(8)_ = 0.9381, *p* = 0.0002; Pearson’s correlation (Extended Data Fig. 3-1*B*); *r*_(8)_ = 0.6308, *p* = 0.0685; Pearson’s correlation (Extended Data Fig. 3-1*D*)]. However, at the single-cell level, we observed substantial populations of EYFP+/tdTomato– and EYFP–/tdTomato+ RGLs and IPCs in all groups (Fig. 3*C–F*), suggesting prevalent mismatch in recombination of the two genes in individual NSPCs.

10.1523/ENEURO.0470-19.2020.f3-1 Extended Data Figure 3-1 Cell-specific fluorescent reporter recombination frequency and correlation. ***A***, Percent of EYFP+ or tdTomato+/GFAP+ RGLs were compared in 3D short, 3D long, and 5D mice. ***B***, Correlation of percent of GFAP+ RGLs that express EYFP and tdTomato in all mice. ***C***, Percent of EYFP+ or tdTomato+ IPCs were compared in 3D short, 3D long, and 5D mice. ***D***, Correlation of percent of Ki67+ IPCs that express EYFP and tdTomato in all mice; *n* = 3 mice per group. Data are shown as mean ± SEM; *p < 0.05, determined by two-way ANOVA (***A***, ***C***) or Pearson’s correlation (***B***, ***D***). *Figure Contributions*: Tyler Joseph Dause ran experiments and analyzed data. Tyler Joseph Dause and Elizabeth Diana Kirby made figures. Download Figure 3-1, TIF file.

**Figure 4.**
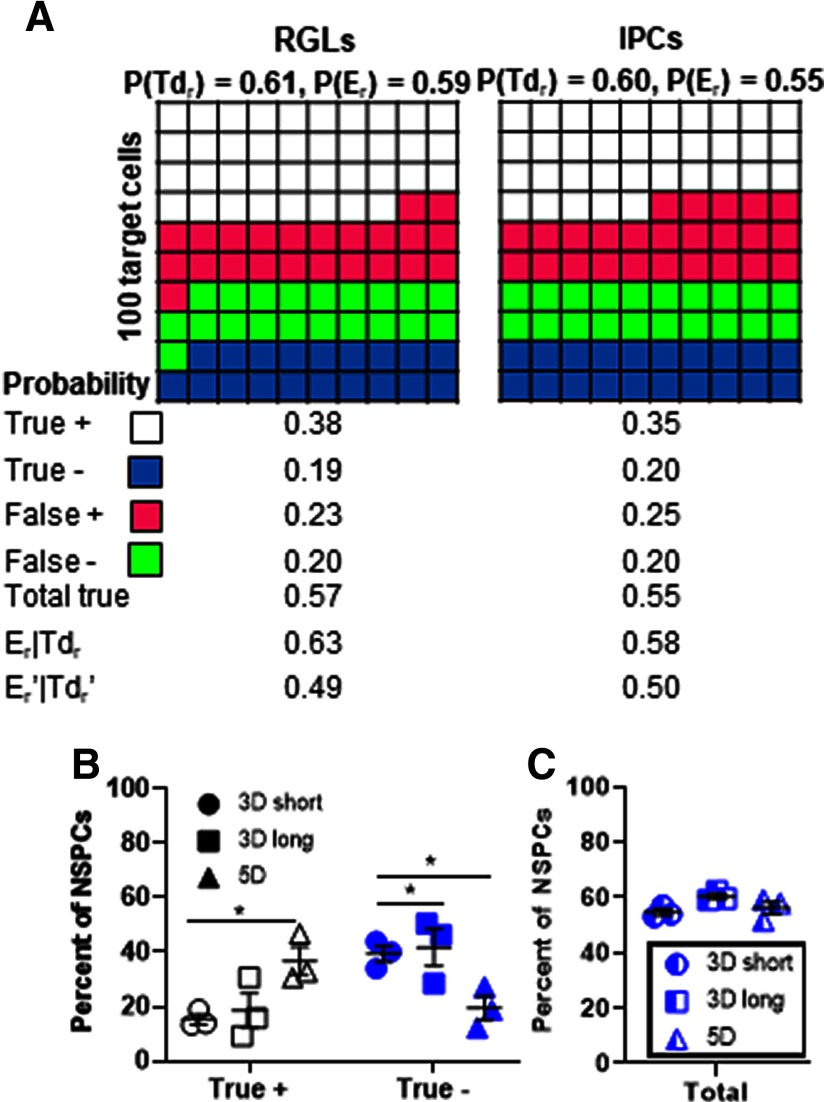
Using expression of one reporter to predict expression of the other results in high false signals similarly across TAM protocols. ***A***, The mean observed recombination frequencies from the 5D TAM group are represented here as reporter expression in 100 hypothetical target cells, either RGLs or IPCs. The probabilities of true and false signals are given as if tdTomato expression is being used to predict EYFP expression. ***B***, The percent of RGLs and IPCs combined (total NSPCs) that show recombination in both reporter genes (true +) or neither (true –) is shown for three TAM groups. ***C***, The total true signal between the three TAM groups is shown; *n* = 3 mice per group. Data shown are mean ± SEM; *p < 0.05 determined by two-way ANOVA. See also Extended Data Figures 4-1, 4-2. *Figure Contributions*: Elizabeth Diana Kirby performed statistical analyses and made figures.

To examine the consequences of the rates of single reporter recombination observed in our in vivo experiments, we applied the reporter expression frequencies observed in the 5D TAM group to a theoretical experiment where tdTomato (Td_r_, equivalent of R_r_ in Fig. 1) is used to predict recombination in the EYFP reporter gene (E_r_, equivalent of T_r_ in Fig. 1). In the 5D TAM group, the conditional probability P(E_r_|Td_r_) was 0.63 or 0.58 for RGLs and IPCs, respectively, meaning that there was a 63% or 58% probability that an RGL or IPC identified as tdTomato+ also showed EYFP expression (Fig. 4*A*). The converse, P(E_r_’|Td_r_’), was 0.49 and 0.50 for RGLs and IPCs, respectively, meaning that identifying an RGL or IPC as tdTomato negative only yielded a 49% or 50% probability that the cell was also EYFP negative (Fig. 4*A*). Comparing true positive and true negative signal in NSPCs across all three TAM protocols revealed that TAM protocol significantly interacted with the type of true signal (positive vs negative; *F*_(2,6)_ = 6.084, *p* = 0.0360; two-way ANOVA; Fig. 4*B*; Extended Data Fig. 4-1*A*). *Post hoc* Tukey’s comparisons revealed that 5D TAM mice showed higher true positive signal than 3D short mice (*p* = 0.0225), but also lower true negative signal than 3D short or 3D long groups (*p* = 0.0328, *p* = 0.0179; Fig. 4*B*). When total true signal was summed, no difference was observed between TAM protocols (*F*_(2,6)_ = 3.201, *p* = 0.1132; one-way ANOVA Fig. 4*C*). Findings were similar when RGLs and IPCs were considered separately (Extended Data Fig. 4-1*B–E*). These findings suggest that despite a wide range of recombination frequencies, none of the tested TAM protocols led to better recombination concordance within single cells than the others.

10.1523/ENEURO.0470-19.2020.f4-1 Extended Data Figure 4-1 Effects of cell type and TAM protocol on accuracy of using expression of one reporter to predict the other. ***A***, The mean observed recombination frequencies from the 3D short (left) and 3D long (right) groups are represented here as reporter expression in 100 hypothetical target cells, either RGLs or IPCs. The probabilities of true and false signals are given as if tdTomato expression is being used to predict EYFP expression. ***B***, The percent of GFAP+ RGL cells that show recombination in both reporter genes (true +) or neither (true –) is shown for three TAM groups. ***C***, The total true signal (+ and –) in GFAP+ RGL cells for the three TAM groups is shown. ***D***, The percent of Ki67+ IPCs that show recombination in both reporter genes (true +) or neither (true –) is shown for three TAM groups. ***E***, The total true signal (+ and –) in Ki67+ IPCs for the three TAM groups is shown; *n* = 3 mice per group. Data shown are mean ± SEM; *p < 0.05, determined by two-way ANOVA. *Figure Contributions*: Tyler Joseph Dause ran experiments and analyzed data. Tyler Joseph Dause and Elizabeth Diana Kirby made figures. Download Figure 4-1, TIF file.

10.1523/ENEURO.0470-19.2020.f4-2 Extended Data Figure 4-2 Fluorescent reporter recombination and colocalization in SVZ NSPCs. ***A***, Immunostaining of EYFP, tdTomato in SVZ NSPCs. Scale bars: 100 μm. Arrowhead = EYFP+/tdTomato– NSPC. Arrow = EYFP–/tdTomato+ NSPC. Chevron = EYFP+/tdTomato+ NSPC. ***B***, Correlation of EYFP+ and tdTomato+ SVZ percent area in all mice. ***C***, Comparison of EYFP+ colocalization in tdTomato+ area to theoretical 100% colocalization. ***D***, Comparison of tdTomato+ colocalization in EYFP+ area to theoretical 100% colocalization; *n* = 9 mice. Data are shown as mean ± SEM; ****p < 0.0001 determined by Pearson’s correlation (***B***) or one-sample *t* test against a theoretical 100% (***C***, ***D***). *Figure Contributions*: Tyler Joseph Dause ran experiments and analyzed data. Tyler Joseph Dause and Elizabeth Diana Kirby made figure. Download Figure 4-2, TIF file.

Although we focus on NSPCs in the SGZ, NSPCs are also found in the SVZ. Similar to the SGZ, in the SVZ, TAM administration induced robust expression of both reporters that was correlated at the population level (*r*_(9)_ = 0.7908, *p* = 0.0077; Pearson’s correlation; Extended Data Fig. 4-2*A*,*B*). However, as with the DG, we found significant divergence of expression at the single-cell level. TdTomato+ colocalization within the EYFP+ area (57%) was significantly less than 100% in the SVZ (*t*_(2)_ = 15.08, *p* < 0.0001, one-sample *t* test comparison to 100%) as was EYFP+ percent area of tdTomato+ area (40%; *t*_(2)_ = 9.115, *p* = 0.0001, one-sample *t* test comparison to 100%; Extended Data Fig. 4-2*C*,*D*). These findings support and extend those from the SGZ by again showing that recombination of one fluorescent reporter gene may not accurately predict recombination of another at the single-cell level in adult NSPCs.

## Discussion

Cell-specific gene manipulation is a powerful tool for investigating complex cellular mechanisms like neurogenesis *in vivo*. Here, we examine a common experimental model to assess cell autonomous function in which Cre-induced recombination of a stop-floxed fluorescent reporter is used to predict recombination of a target gene at the single-cell level. The reliability of this model is contingent on the assumption that transiently-activated Cre induces independent recombination events equivalently within a single cell. We first examined this experimental paradigm with probabilistic calculations, which revealed that mild variation in recombination efficiencies and discordance between recombination in a fluorescent reporter gene and a target gene could lead to substantial error in identifying cells with target gene recombination. These data led us to examine the accuracy of this methodology in a mouse model. In our model, we quantified TAM-induced, Cre-dependent recombination of two stop-floxed fluorescent reporter constructs in adult NSPCs. Our findings revealed that, while both reporters were highly expressed in NSPCs and global recombination rates of the two reporters were correlated, expression of either fluorescent reporter was an inaccurate predictor of expression of the other in individual NSPCs. Although different Cre drivers and different reporters may yield different results from those obtained here, our data suggest that it may not be reliable to assume that recombination of one gene is predictive of recombination of a second gene within single cells using TAM-sensitive Cre recombinases to drive recombination.

We began our investigation by modeling the theoretical accuracy of using Cre-induced recombination of one gene to predict recombination in another gene. In our model, stop-floxed fluorescent reporter gene (Gene R) expression is used as a marker of target gene (Gene T) recombination for analyses of cell autonomous target gene function. Using reasonable experimental expectations in a generic experimental design, we found that presence of a reporter protein R could be a poor predictor of target gene recombination. Even in a case where maximal concordance of recombination occurs, if a 2-fold difference in overall recombination rates were present, reporter presence would accurately predict target gene recombination in only 50% of cells. A previous study using three separate stop-floxed reporters each combined with the same NestinCreER^T2^ drivers revealed recombination efficiencies ranging over 10-fold (Sun et al., 2014), suggesting that our allowance of a 2-fold difference in efficiency is well within the experimentally probable range. Our theoretical predictions therefore suggest that using stop-floxed fluorescent reporter proteins to identify target gene recombined cells could yield false signal at a high frequency even if the only source of error were difference in overall recombination efficiency.

Next, we examined the results of our theoretical models in an *in vivo* experimental paradigm where we used two separate fluorescent reporters as indicators of two possible recombination events within a single cell. We used a highly-cited, NSPC-specific and efficient NestinCreER^T2^ line to drive recombination of two highly-cited stop-floxed fluorescent reporters. In these mice, we found that presence of one reporter accurately predicted presence of the other reporter in only 50–60% of NSPCs. While different reporters and different Cre-drivers may reveal different results, our data suggest that the assumption that two, independent recombination events show high concordance within a cell may be false, even in mouse lines used in hundreds of studies.

The likelihood of Cre-mediated recombination events can be influenced by several factors: the paradigm of Cre induction, features of the Cre recombinase, and features of the loxP-flanked genes. First, Cre induction and resulting loxP recombination can depend on the dose, frequency and route of TAM administration, as well as the delay between TAM and tissue harvest. For example, here we show that five TAM injections significantly increased global fluorescent reporter recombination rates compared with three TAM injections, as one would expect. However, of three different TAM injection protocols that varied in number of injections and delay before harvest, we found that no one of the protocols led to higher concordance in recombination (or lack of recombination) in the two reporter genes than the others. Nonetheless, other dosing schedules or routes (such as orally) may yield different results.

Features of the Cre recombinase can also impact likelihood of loxP recombination. Previous work shows that efficiency of different CreER^T2^ lines at driving recombination events can vary substantially (Sun et al., 2014). These differences may be due to the sequence of the recombinase gene itself or due to its insertion location in the genome. Use of inducible Cre, as opposed to a constitutively active form of Cre, also likely decreases recombination efficiency. The NestinCreER^T2^ line used in the present study is one of the lower efficiency, inducible NSPC-targeted Cre lines of those available (Sun et al., 2014). A NestinCreER^T2^ line with higher efficiency would likely show greater concordance in recombination events within single cells. However, if the goal of a study is to compare recombined and non-recombined cells, driving recombination to near 100%, while it would yield better concordance rates across genes, would eliminate the non-recombined cell population of comparison. The importance of recombination specificity also must be considered when selecting a Cre-driver line. For example, although the NestinCreER^T2^ line used here has low efficiency, it is also more specific to NSPCs than the comparable lines with higher efficiency (Sun et al., 2014).

The features of loxP flanked constructs can also impact the likelihood of Cre-lox recombination. In the present study, paired fluorescent reporter genes were located in identical Rosa loci, making them more similar than most reporter-target combinations. However, the tdTomato construct contained a CAG promotor that the EYFP construct lacked. The distance between loxP sites also differed, with the tdTomato construct having 0.9 kb between loxP sites while the EYFP construct contained 2.7 kb between loxP sites. The above factors could lead to preferential recombination of tdTomato expression over EYFP, which we observed signs of in the quantification of fluorescent area. However, manual cell counts did not show greater detection of one reporter over the other, and both EYFP+/tdTomato– and EYFP–/tdTomato+ single cells were found, suggesting that our findings of poor concordance are not driven solely by lower recombination rates in one reporter gene compared with the other. In most experimental paradigms designed to test cell autonomous gene function, expression of a stop-floxed fluorescent reporter is assumed to imply recombination of a separate target gene, which has a different promoter, different loxP site separation and different gene sequence than the reporter construct. Therefore, while the two reporters used here are not identical, they share many similarities that should drive single-cell concordance rates to be higher than in a typical experiment where target gene and reporter are less related. Our findings therefore suggest that even with similar overall recombination rates and similar genetic locations, there can be substantial divergence in recombination events within a single cell.

A separate issue from gene recombination is detection of recombination. Fluorescent reporter expression, although present, may not reach the threshold for detection in every cell. To combat this, we performed both manual and automated data analyses, which both yielded similar findings. However, using more sensitive microscopy methods for detecting fluorescent markers may reveal a higher percentage of overlap in reporter expression. The relative intensity of EYFP signal versus tdTomato signal may also differ, driving different detection rates of each reporter. Antibodies against EYFP and tdTomato were used in the present work to increase detection of both reporters and only a slight increase in tdTomato detection was found over EYFP in data derived from fluorescent area. The concern of fluorescence detection would also apply to empirical studies of gene recombination function and may be a source of error there as well.

A relevant question raised by our theoretical and experimental results is how much error is acceptable in studies of cell autonomous gene function. Using reporter protein presence (or absence) to define cells as target gene recombined (or intact) is effectively dividing cells in to separate experimental groups. We therefore suggest that the error acceptable for reporter and target gene recombination concordance should be similar to error researchers would deem acceptable in identifying animals in different experimental groups. When considering the standard for error used in biological statistics, we suggest that a reasonable experimental expectation of this model would be gene recombination concordance of at least 95%. The observed 50–60% accuracy of one reporter predicting recombination of another reporter found in the present study falls far short of this threshold, and also is likely much lower than what most investigators would consider acceptable error in identifying treatment groups in a randomized study.

Given the large number of CreER lines, fluorescent reporters, and individual floxed genes available, it may be impossible to determine how widely the present findings apply. Different Cre driver-reporter gene-target gene combinations could substantially differ from the present results, yielding more or less reliable concordance in recombination between separate genes. Ideally, the concordance of target gene and reporter gene recombination would be tested in each specific transgenic model where it is being applied for study of cell autonomous gene recombination. This process would require some form of alternative verification of target gene recombination such as immunohistochemistry or in situ hybridization. However, stop-floxed reporters are typically used precisely because other methods of identifying target gene-recombined cells are lacking. We therefore suggest that these results present a cautionary note and that given the potential for error in this method, alternative methods of studying cell autonomous gene function should be considered.

There are several alternative methods for identifying the effects of cell autonomous gene manipulation. One paradigm that still uses inducible Cre is to integrate fluorescent reporters in the target genomic sequence. Such models will likely require creation of new transgenic mice for many target genes, as few existing models include linked reporters in this fashion. An additional alternative method is to deliver transgenes plus cleavably-linked fluorescent reporters using viral vectors that target expression to specific cell populations based on viral serotype or cell-specific promoters. For example, certain adeno-associated viruses show preference for infecting specific NSPC subclasses and can be used to manipulate gene function in adulthood after stereotaxic delivery (Crowther et al., 2018).

In addition to tracking recombination in single cells, stop-floxed reporters are also frequently used to identify the cell population targeted by a Cre-driver line (i.e., population specificity), as well as overall recombination efficiencies. We found that both tdTomato and EYFP expression were similarly concentrated in adult NSPCs, suggesting that cell population specificity of recombination was similar regardless of reporter. We also found a strong correlation between recombination efficiency of the two reporters in individual mice, suggesting that, at least in this case, reporters could reliably predict high-recombined subjects versus low-recombined subjects. However, it is unlikely that absolute recombination efficiencies can be extrapolated across genes due to the well-documented differences in loxP recombination probability across genomic loci (Long and Rossi, 2009; Gray et al., 2017).

Our findings do not necessarily undermine previous studies which have identified cell autonomous gene functions using stop-floxed reporters as a marker of target gene recombined cells (Zhou et al., 2018; Zimmermann et al., 2018; Franz et al., 2019; Zhang et al., 2019). Aside from the possibility that other reporter-target combinations show better recombination concordance than the two genes used in this study, if target gene recombination has a large cell-autonomous effect, it may still be detectable in models with suboptimal reporter-target recombination concordance due to the subpopulation of cells in which fluorescent protein presence is a true signal. False negative and false positive signals like those observed in this model, if present in other models, would be most likely to obscure smaller cell autonomous effects and lead to false negative findings when cell autonomous gene functions are in fact present.

In summary, our findings suggest that models of inducible gene manipulation combined with a ubiquitously-expressed stop-floxed fluorescent reporter could be unreliable in their ability to identify cell autonomous effects at a single-cell level *in vivo*. We support this finding with theoretical probability estimates of a generic Cre-lox experiment and experimental data from one example Cre-lox system. Future work is necessary to create reliable and cost-effective models that can be easily applied to the study of cell-autonomous effects across many target genes. Such models will be imperative for studying molecular mediators of complex cellular processes such as adult neurogenesis.

## Synthesis

Reviewing Editor: Dritan Agalliu, Columbia University Medical Center Decisions are customarily a result of the Reviewing Editor and the peer reviewers coming together and discussing their recommendations until a consensus is reached. When revisions are invited, a fact-based synthesis statement explaining their decision and outlining what is needed to prepare a revision will be listed below. The following reviewer(s) agreed to reveal their identity: Jason Snyder, Grant MacGregor.

This is a short but interesting and important paper on the efficacy of cre-mediated recombination in inducible systems. The authors show that a widely-used NestinCreER mouse, where Cre is inducibly activated upon administering tamoxifen, leads to recombination of a EYFP reporter in some stem cells, a tdTomato reporter in other cells, and both reporters in another population of cells. This directly challenges the assumption that recombination is equivalent at two different target sites in a given cell, and identifies an important confound for studies that use cre recombination to both modify one gene and induce expression of a reporter in the same cell. The report will raise awareness within the broader community of the potential problems associated with inferring activation of a LSL-reporter gene with loxP recombination at another site within the genome in a cell.

Reviewer # 1

Minor concerns:

1) I do not see any critical flaws but do think there are a few issues to address regarding the possibility that differences may not be due to recombination variability but rather due to differential ability to measure the two reporters equivalently. I do not have emprical evidence for this but I believe, have heard, that the EYFP signal often used in these Nestin CreER mice is rather weak whereas the tdTomato mice give a very strong signal. I therefore wonder whether this could bias the results. Eg in Fig 2 it looks like there is a systematic difference where EYFP is consistently lower than Tomato. The use of automated quantification methods of area that is positive for reporter signals doesn’t help (eg what thresholding may introduce biases where weakly positive cells are excluded). Might this also be more of an issue for younger cells that may not have “ramped” up their reporter expression levels? Notably, these concerns are somewhat tempered by the data in Fig 3, which is based on manual cell counts. Also, the images in the provided manuscript are low resolution so it is difficult to assess the likelihood that visualization issues may be at play.

2) What are the density units for Fig 3C,E?

3) What was the sampling procedure for the cell counts?

Reviewer # 2

The study addresses the question whether Cre-mediated activation of a reporter gene in mice can reliably infer the status of Cre-mediated recombination at additional sites within the genome of the same cell, in this case within adult neuronal stem and progenitor cells. This method is often employed when no antibody is available to analyze the status by IHC/IF of a gene product expected to be depleted following Cre-mediated recombination of a cKO floxed allele. This is an important issue that is likely to be of interest to a wide range of researchers using inducible Cre-lox systems in their studies.

Several factors can affect the outcome of an experiment to analyze inducible Cre-mediated recombination between loxP sites that removes a transcriptional termination signal. These include (i) the specific CreERT2 transgene or KI allele being used, and the level of expression of CreERT2, (ii) the dose, frequency and route of administration of Tam, (iii) the time between administration of Tam and analysis of recombination, (iv) the promoter driving expression of the fluorescent reporter gene, (v) the distance between the loxP sites in the LSL sequence, (vi) the genomic region containing the LSL-reporter construct and (vii) the methods used to process tissue and quantify reporter gene expression. Given the large number of CreERT, LSL reporter and individual floxed gene alleles available, it is challenging to determine the potential impact of findings regarding efficiency of Cre activity in any single study to another.

Although unlikely to reflect real-world application of the method, one way to investigate the maximum likelihood that a specific Cre-ERT2 expressing locus could mediate recombination at two separate LSL fluorescent reporters is to combine a transgene etc that expresses CreERT2 at a high level, with LSL-reporter genes in which only the reporter gene sequence has been varied (e.g. comparing Ai3 (EYFP) with Ai9 (tdTomato) reporters (Madisen et al, 2010). The authors seem to allude to doing this on page 10 of the manuscript where they state “The floxed sequences used, Rosa-stop-floxed-EYFP and Rosastop-floxed-tdTomato genes, are both inserted in the Rosa locus, a similarity intended to equalize both the Cre-accessibility of the two genes and the promoter activity driving their expression, thereby maximizing the likelihood of homozygous recombination within single Nestin-expressing cells after TAM exposure”. This is also suggested by the graphic in Fig 2A. However, both the statement and Fig2A appear somewhat inaccurate and the methods and reagents used seem likely to produce outcomes that could underestimate the potential utility of LSL-reporter genes as a read out of CreERT2 activity.

In the ROSA-stop-floxed EYFP (Jax stock # 006148; Srinivas et al 2001), transcription of the EYFP coding sequence is mediated by the ROSA26 lncRNA promoter. Although this promoter is also present in the ROSA-stop-floxed-tdTomato (Jax stock # 007909; Madisen et al 2010) Ai9 line, expression of tdTomato is mediated by the considerably stronger hybrid CMV enhancer chicken beta-actin (CAG) promoter. This results in considerably different expression levels from each reporter gene - e.g. see Fig 2 in Madisen et al 2010). As reported (Sun et al, 2014), the sensitivity of the reporter line can affect the apparent expression pattern observed for a cre driver.

A second difference that could impact the outcome of the experiment is the difference in the distance between the loxP sequences in the two reporter lines used. Fig 2A suggests the LSL sequences are similar. In fact, the distance between the loxP sites in each LSL is very different - 0.9kb for the tdTomato construct but 2.7kb for the EYFP construct. This reviewer is unaware of a report in the literature that performed a systematic analysis on the effect of increasing the distance between two loxP sites in the 0.5 - 50 kb range at different loci in the genome. However, based on Sun et al, 2014 and Morozov (Curr Protoc Neurosci Chapter 4: Unit 4.31 2008), it seems plausible that recombination frequency between loxP site in cis decreases with increasing distance between them. Reduced cre-mediated efficiency of removal of the stop signals in the longer (EYFP) construct might also be consistent with the finding in the present study that tdTomato-positive cells were less likely to be EYFP-positive than the converse.

The authors do not state the type of camera used to acquire the fluorescent signals and its sensitivity. Another possibility is that a higher-sensitivity method (e.g. using GaAsP detectors in photon counting mode) may increase the number of cells that appear to be expressing both reporters.

Hence, the combination of the relatively weakly expressing nestin-CreERT2 line K transgenic line used, plus the reporter constructs and the method of detection might have produced an outcome that under-estimates the ability of CreERT2 to mediate recombination at two separate sets of loxP sites within the same cell. In this light, I would suggest that the hypothesis that the authors’ results “represent a best-case scenario” for concordance between reporter expression and target recombination (p16, line 12) may not hold true.

Despite these issues, there is still merit to the study. However, the report would benefit from inclusion of a balanced discussion of the above issues, including that the authors’ findings are made using a specific set of reagents and methods that may not apply to other studies.

The authors could also improve the balance in their report by tempering statements that could be interpreted as casting doubt on the utility of LSL-reporter genes under any circumstance. For example, in the Abstract, in the statement “This poor concordance in floxed sequence recombination across genes implies that use of stop-floxed reporters to investigate cell autonomous gene function is likely to lead to false conclusions”, “Implies” could be substituted with ‘suggests’ while “is likely to” could be substituted with ‘could’. Similar modification of related statements should also be made throughout the text.

Typographical errors

Page 16, line 17 - “in tact” should be ‘intact’

Page 35, Figure Extended Data 1-1 - “Contraint” should be “Constraint”.

## Author Response

We would like to thank the editor and reviewers for their time and consideration of our manuscript. We have addressed each reviewer concern below and in the attached, improved version of the manuscript.

Reviewer # 1

Minor concerns:

1) I do not see any critical flaws but do think there are a few issues to address regarding the possibility that differences may not be due to recombination variability but rather due to differential ability to measure the two reporters equivalently. I do not have emprical evidence for this but I believe, have heard, that the EYFP signal often used in these Nestin CreER mice is rather weak whereas the tdTomato mice give a very strong signal. I therefore wonder whether this could bias the results. Eg in Fig 2 it looks like there is a systematic difference where EYFP is consistently lower than Tomato. The use of automated quantification methods of area that is positive for reporter signals doesn't help (eg what thresholding may introduce biases where weakly positive cells are excluded). Might this also be more of an issue for younger cells that may not have “ramped” up their reporter expression levels? Notably, these concerns are somewhat tempered by the data in Fig 3, which is based on manual cell counts. Also, the images in the provided manuscript are low resolution so it is difficult to assess the likelihood that visualization issues may be at play.

In response to the concern that one fluorescent reporter may be more easily detectable than another, we have added more discussion about the role that signal detection plays in evaluating Cre-mediated recombination in the discussion. In addition, we make it more clear that we used antibodies against GFP and mCherry to maximize signal detection. As reviewer 1 suggests is the case, we have indeed observed that endogenous eYFP signal is weak. This is why we included antibodies to amplify signal substantially.

In response to the concern about image resolution, figures were submitted as a part of the submission pdf according to eNeuro guidelines, which compressed the images substantially. Upon resubmission, high resolution TIFFs of figures/images were attached to improve image resolution.

2) What are the density units for Fig 3C,E?

Density units were added to Figure 3C,E: Density represented as cells per area, scale x 10-4.

3) What was the sampling procedure for the cell counts?

The sampling procedure for cell counts was added to our methods section under “NSPC Identification and Manual Cell Counts”. All NSPCs were identified and counted in each section.

Reviewer # 2

The study addresses the question whether Cre-mediated activation of a reporter gene in mice can reliably infer the status of Cre-mediated recombination at additional sites within the genome of the same cell, in this case within adult neuronal stem and progenitor cells. This method is often employed when no antibody is available to analyze the status by IHC/IF of a gene product expected to be depleted following Cre-mediated recombination of a cKO floxed allele. This is an important issue that is likely to be of interest to a wide range of researchers using inducible Cre-lox systems in their studies.

Several factors can affect the outcome of an experiment to analyze inducible Cre-mediated recombination between loxP sites that removes a transcriptional termination signal. These include (i) the specific CreERT2 transgene or KI allele being used, and the level of expression of CreERT2, (ii) the dose, frequency and route of administration of Tam, (iii) the time between administration of Tam and analysis of recombination, (iv) the promoter driving expression of the fluorescent reporter gene, (v) the distance between the loxP sites in the LSL sequence, (vi) the genomic region containing the LSL-reporter construct and (vii) the methods used to process tissue and quantify reporter gene expression. Given the large number of CreERT, LSL reporter and individual floxed gene alleles available, it is challenging to determine the potential impact of findings regarding efficiency of Cre activity in any single study to another.

We agree. There are many factors can affect the outcome of our experiments. Importantly, these concerns also generally apply to the experiments that we are modeling and therefore represent general concerns when using stop-floxed reporters to draw conclusions about Cre-mediated gene recombination. We have substantially expanded our discussion of the issues indicated by Reviewer 2 in the Discussion.

Although unlikely to reflect real-world application of the method, one way to investigate the maximum likelihood that a specific Cre-ERT2 expressing locus could mediate recombination at two separate LSL fluorescent reporters is to combine a transgene etc that expresses CreERT2 at a high level, with LSL-reporter genes in which only the reporter gene sequence has been varied (e.g. comparing Ai3 (EYFP) with Ai9 (tdTomato) reporters (Madisen et al, 2010). The authors seem to allude to doing this on page 10 of the manuscript where they state “The floxed sequences used, Rosa-stop-floxed-EYFP and Rosastop-floxed-tdTomato genes, are both inserted in the Rosa locus, a similarity intended to equalize both the Cre-accessibility of the two genes and the promoter activity driving their expression, thereby maximizing the likelihood of homozygous recombination within single Nestin-expressing cells after TAM exposure”. This is also suggested by the graphic in Fig 2A. However, both the statement and Fig2A appear somewhat inaccurate and the methods and reagents used seem likely to produce outcomes that could underestimate the potential utility of LSL-reporter genes as a read out of CreERT2 activity.

In the ROSA-stop-floxed EYFP (Jax stock # 006148; Srinivas et al 2001), transcription of the EYFP coding sequence is mediated by the ROSA26 lncRNA promoter. Although this promoter is also present in the ROSA-stop-floxed-tdTomato Jax stock # 006148; Madisen et al 2010) Ai9 line, expression of tdTomato is mediated by the considerably stronger hybrid CMV enhancer chicken beta-actin (CAG) promoter. This results in considerably different expression levels from each reporter gene - e.g. see Fig 2 in Madisen et al 2010). As reported (Sun et al, 2014), the sensitivity of the reporter line can affect the apparent expression pattern observed for a cre driver.

A second difference that could impact the outcome of the experiment is the difference in the distance between the loxP sequences in the two reporter lines used. Fig 2A suggests the LSL sequences are similar. In fact, the distance between the loxP sites in each LSL is very different - 0.9kb for the tdTomato construct but 2.7kb for the EYFP construct. This reviewer is unaware of a report in the literature that performed a systematic analysis on the effect of increasing the distance between two loxP sites in the 0.5 - 50 kb range at different loci in the genome. However, based on Sun et al, 2014 and Morozov (Curr Protoc Neurosci Chapter 4: Unit 4.31 2008), it seems plausible that recombination frequency between loxP site in cis decreases with increasing distance between them. Reduced cre-mediated efficiency of removal of the stop signals in the longer (EYFP) construct might also be consistent with the finding in the present study that tdTomato-positive cells were less likely to be EYFP-positive than the converse.

We agree with reviewer 2 that our statements and figures did not accurately represent the differences between the two fluorescent reporters, including promoters used and LSL distance. In response to this concern we added more genetic information about our stop-floxed fluorescent reporters to the Results and Discussion sections. Additionally, we added discussion about how differences in expression of the fluorescent reporters may impact our data in the Discussion. However, we do not think that these differences undercut the value of our data. Our data are meant to show results from an example model of a common experimental system. In the common experimental system, Cre-mediated recombination of a stop-floxed reporter is used to infer recombination in a separate gene that likely differs from the reporter construct in many ways, including those highlighted by reviewer 2. While we agree that our claim that our example represents a best-case scenario was an overstatement (and that has been corrected), we still think our example paradigm does not necessarily provide an underestimate due to the differences in genetic construct. The differences between reporter sequences present in our model are within a reasonable range for the reporter-target systems we are modeling, in our opinion. We have discussed these issues in much greater detail in the discussion in response to this concern.

The authors do not state the type of camera used to acquire the fluorescent signals and its sensitivity. Another possibility is that a higher-sensitivity method (e.g. using GaAsP detectors in photon counting mode) may increase the number of cells that appear to be expressing both reporters.

We agree a more powerful microscope/camera may have increased our ability to detect fluorescent signals and their overlap. First, we added information about our camera to the Methods section to clarify our image acquisition methodology. Second, while a more sensitive microscope or camera may have increased our ability to detect both fluorescent reporters, possibly increasing the number of cells that appear to be expressing both reporters, our tools for fluorescence imaging are similar to those used in most experimental methodologies. In support of the sensitivity of our microscopy methods being similar to that widely applied to assess Cre-recombination, we show that recombination efficiency of both reporters (i.e. percent of NSPCs expressing reporters) is similar to that reported in previous studies (see Fig 3-1A-D). As such, we believe that concerns about fluorescence detection would apply to most studies of gene recombination and have added discussion of this as a potential limitation in the Discussion section.

Hence, the combination of the relatively weakly expressing nestin-CreERT2 line K transgenic line used, plus the reporter constructs and the method of detection might have produced an outcome that under-estimates the ability of CreERT2 to mediate recombination at two separate sets of loxP sites within the same cell. In this light, I would suggest that the hypothesis that the authors' results “represent a best-case scenario” for concordance between reporter expression and target recombination (p16, line 12) may not hold true.

We agree with Reviewer 2's concerns (addressed above) and our description of our model representing a “best-case scenario” has been removed.

Despite these issues, there is still merit to the study. However, the report would benefit from inclusion of a balanced discussion of the above issues, including that the authors' findings are made using a specific set of reagents and methods that may not apply to other studies.

Discussion about the applicability of our methods has been added to the Discussion section, as well as balanced discussion of the above issues (above).

The authors could also improve the balance in their report by tempering statements that could be interpreted as casting doubt on the utility of LSL-reporter genes under any circumstance. For example, in the Abstract, in the statement “This poor concordance in floxed sequence recombination across genes implies that use of stop-floxed reporters to investigate cell autonomous gene function is likely to lead to false conclusions”, “Implies” could be substituted with ‘suggests’ while “is likely to” could be substituted with ‘could’. Similar modification of related statements should also be made throughout the text.

The wording of the manuscript has been tempered in response to Reviewer 2's concerns. We agree that our wording was too strong. We think a better description of the implications of the findings is that in most models using stop-floxed reporters, no data is available about the single cell concordance in recombination between reporter detection and target recombination. Our data shows that this concordance can be quite low and as a result, it may be unwise to assume a specific reporter-target system is useful in this type of experiment without further evidence to support its accuracy. This point has been added to the discussion more clearly.

Typographical errors

Page 16, line 17 - “in tact” should be ‘intact’

Page 35, Figure Extended Data 1-1 - “Contraint” should be ‘Constraint’.

Typographical errors were addressed and corrected.
