# Supplementary material for: Poor Concordance of Floxed Sequence Recombination in Single Neural Stem Cells: Implications for Cell Autonomous Studies
Source: eNeuro. 2020 Mar 13;7(2):ENEURO.0470-19.2020. doi: 10.1523/ENEURO.0470-19.2020 (PMC7086402; doi:10.1523/ENEURO.0470-19.2020)
Supplement: Supplementary Extended Data Table 2-1 — Main Figures Raw Data. Download Table 2-1, DOCX file. [file enu-eN-TNC-0470-19-s07.docx]

**Extended Data Table 2-1: Main Figures Raw Data**

| EYFP and tdTomato DG Percent area Comparison | | | | | | | | | | |
| --- | --- | --- | --- | --- | --- | --- | --- | --- | --- | --- |
| Figure | TAM Administration | n | EYFP Mean | Standard Error | tdTomato Mean | Standard Error |  |  |  |  |
| 2E,F | 3D Short | 3 | 0.1442 | 0.01713 | 0.08112 | 0.01544 |  |  |  |  |
| 2E,F | 3D Long | 3 | 0.147 | 0.02412 | 0.08149 | 0.01565 |  |  |  |  |
| 2E,F | 5D | 3 | 0.2736 | 0.05423 | 0.2948 | 0.03554 |  |  |  |  |
|  |  |  |  |  |  |  |  |  |  |  |
| EYFP+ Percent Area Colocalization with tdTomato+ Area | | | | | | | | | | |
| Figure | TAM Administration | n | Colocalized Area | Standard Error |  |  |  |  |  |  |
| 2H | 3D Short | 3 | 15.84 | 2.346 |  |  |  |  |  |  |
| 2H | 3D Long | 3 | 21.77 | 5.947 |  |  |  |  |  |  |
| 2H | 5D | 3 | 36.71 | 2.654 |  |  |  |  |  |  |
|  |  |  |  |  |  |  |  |  |  |  |
| tdTomato+ Percent Area Colocalization with EYFP+ Area | | | | | | | | | | |
| Figure | TAM Administration | n | Colocalized Area | Standard Error |  |  |  |  |  |  |
| 2I | 3D Short | 3 | 25.14 | 0.6255 |  |  |  |  |  |  |
| 2I | 3D Long | 3 | 33.22 | 6.42 |  |  |  |  |  |  |
| 2I | 5D | 3 | 36.3 | 5.654 |  |  |  |  |  |  |
|  |  |  |  |  |  |  |  |  |  |  |
| DG RGL Densities | | | | | | | | | | |
| Figure | TAM Administration | n | EYFP Only Mean | EYFP Only Standard Error | tdTomato Only Mean | tdTomato Only Standard Error | Both Mean | Both Standard Error | Neither Mean | Neither Standard Error |
| 3B | 3D Short | 3 | 0.00015 | 2.42E-05 | 0.00012 | 1.48E-05 | 9.00E-05 | 2.63E-05 | 0.00027 | 4.80E-05 |
| 3B | 3D Long | 3 | 0.00015 | 3.48E-05 | 0.00012 | 7.81E-05 | 0.00011 | 3.18E-05 | 0.00031 | 4.18E-05 |
| 3B | 5D | 3 | 0.0002 | 8.63E-05 | 0.00022 | 1.56E-05 | 0.00039 | 0.00011 | 0.00018 | 3.41E-05 |
|  |  |  |  |  |  |  |  |  |  |  |
| DG RGL Percentages | | | | | | | | | | |
| Figure | TAM Administration | n | EYFP Only Mean | EYFP Only Standard Error | tdTomato Only Mean | tdTomato Only Standard Error | Both Mean | Both Standard Error | Neither Mean | Neither Standard Error |
| 3C | 3D Short | 3 | 23.76 | 3.389 | 19.03 | 2.496 | 14.71 | 5.241 | 42.49 | 5.636 |
| 3C | 3D Long | 3 | 22.21 | 0.097 | 17.26 | 1.426 | 15.85 | 5.037 | 44.69 | 5.241 |
| 3C | 5D | 3 | 20.4 | 1.762 | 22.46 | 1.15 | 38.33 | 6.566 | 18.8 | 4.559 |
|  |  |  |  |  |  |  |  |  |  |  |
| DG Progenitor Densities | | | | | | | | | | |
| Figure | TAM Administration | n | EYFP Only Mean | EYFP Only Standard Error | tdTomato Only Mean | tdTomato Only Standard Error | Both Mean | Both Standard Error | Neither Mean | Neither Standard Error |
| 3E | 3D Short | 3 | 0.00011 | 3.00E-05 | 0.00016 | 3.53E-05 | 8.67E-05 | 3.84E-05 | 0.00021 | 6.43E-05 |
| 3E | 3D Long | 3 | 8.06E-05 | 2.62E-05 | 0.0001 | 1.36E-05 | 0.00011 | 4.37E-05 | 0.00019 | 4.16E-05 |
| 3E | 5D | 3 | 0.00012 | 4.50E-05 | 0.00013 | 2.66E-05 | 0.0002 | 5.00E-05 | 0.00011 | 3.08E-05 |
|  |  |  |  |  |  |  |  |  |  |  |
| DG Progenitor Percentages | | | | | | | | | | |
| Figure | TAM Administration | n | EYFP Only Mean | EYFP Only Standard Error | tdTomato Only Mean | tdTomato Only Standard Error | Both Mean | Both Standard Error | Neither Mean | Neither Standard Error |
| 3F | 3D Short | 3 | 20.17 | 0.8351 | 29.11 | 2.765 | 13.45 | 2.997 | 37.27 | 1.434 |
| 3F | 3D Long | 3 | 19.31 | 2.006 | 20.91 | 2.265 | 22.27 | 8.225 | 37.51 | 8.63 |
| 3F | 5D | 3 | 20.05 | 4.321 | 25.32 | 7.893 | 34.72 | 2.792 | 19.91 | 5.887 |
|  |  |  |  |  |  |  |  |  |  |  |
|  |  |  |  |  |  |  |  |  |  |  |
| DG Percent of NSPCs with True+ Signal | | | | | | | | | | |
| Figure | TAM Administration | n | NSPCs Mean True + Percent | SE |  |  |  |  |  |  |
| 4B | 3D Short | 3 | 15.33 | 1.843 |  |  |  |  |  |  |
| 4B | 3D Long | 3 | 18.54 | 6.334 |  |  |  |  |  |  |
| 4B | 5D | 3 | 36.74 | 6.334 |  |  |  |  |  |  |
|  |  |  |  |  |  |  |  |  |  |  |
| DG Percent of NSPCs with True- Signal | | | | | | | | | | |
| Figure | TAM Administration | n | NSPCs Mean True - Percent | SE |  |  |  |  |  |  |
| 4B | 3D Short | 3 | 39.34 | 2.886 |  |  |  |  |  |  |
| 4B | 3D Long | 3 | 41.66 | 6.739 |  |  |  |  |  |  |
| 4B | 5D | 3 | 19.37 | 4.32 |  |  |  |  |  |  |
|  |  |  |  |  |  |  |  |  |  |  |
| DG Percent of NSPCs with True+/- Signal | | | | | | | | | | |
| Figure | TAM Administration | n | NSPCs Mean True +/- Percent | SE |  |  |  |  |  |  |
| 4C | 3D Short | 3 | 54.66 | 1.239 |  |  |  |  |  |  |
| 4C | 3D Long | 3 | 60.21 | 0.9257 |  |  |  |  |  |  |
| 4C | 5D | 3 | 56.12 | 2.312 |  |  |  |  |  |  |
