# Supplementary material for: Poor Concordance of Floxed Sequence Recombination in Single Neural Stem Cells: Implications for Cell Autonomous Studies
Source: eNeuro. 2020 Mar 13;7(2):ENEURO.0470-19.2020. doi: 10.1523/ENEURO.0470-19.2020 (PMC7086402; doi:10.1523/ENEURO.0470-19.2020)
Supplement: Supplementary Extended Data Table 2-2 — Extended Data Figures Raw Data and Statistics. Download Table 2-2, DOCX file. [file enu-eN-TNC-0470-19-s08.docx]

**Extended Data Table 2-2 Extended Data Figures Raw Data and Statistics**

| DG EYFP+ and tfTomato+ RGL % | | | | | | | | | | | |
| --- | --- | --- | --- | --- | --- | --- | --- | --- | --- | --- | --- |
| Figure | TAM | n | EYFP Mean | Standard Error | tdTomato Mean | Standard Error | test | Comparison | statistic | p value | Significant? |
| 3-1A | 3D Short | 3 | 38.48 | 4.147 | 33.74 | 7.564 | 2-way ANOVA | TAM x Reporter | F (2, 6) = 1.216 | P=0.3602 | No |
| 3-1A | 3D Long | 3 | 38.06 | 5.125 | 33.11 | 5.146 |  | TAM | F (2, 6) = 6.678 | P=0.0298 | Yes * |
| 3-1A | 5D | 3 | 58.73 | 5.55 | 60.79 | 5.747 |  | Reporter | F (1, 6) = 1.484 | P=0.2689 | No |
|  |  |  |  |  |  |  |  | Subject | F (6, 6) = 8.742 | P=0.0092 | Yes ** |
|  |  |  |  |  |  |  | Post-hoc | Comparisons | Adjusted p value | Significant? |  |
|  |  |  |  |  |  |  | Tukey's multiple comparisons | 3D short vs. 3D long | p = 0.9973 | No |  |
|  |  |  |  |  |  |  |  | 3D short vs. 5D | p = 0.0464 | Yes * |  |
|  |  |  |  |  |  |  |  | 3D long vs. 5D | p = 0.0426 | Yes * |  |
|  | | | | | | | | | | | |
| EYFP and tdTomato DG RGL Density Correlation | | | | | | | | | | | |
| Figure | TAM | n | test | statistic | p value | Significant? |  |  |  |  |  |
| 3-1B | All | 9 | Pearson's Correlation | r(8) = 0.9381 | p = 0.0002 | Yes *** |  |  |  |  |  |
|  | | | | | | | | | | | |
| DG EYFP+ and tdTomato+ Progenitor % | | | | | | | | | | | |
| Figure | TAM | n | EYFP Mean | Standard Error | tdTomato Mean | Standard Error | test | Comparison | statistic | p value | Significant? |
| 3-1C | 3D Short | 3 | 33.62 | 2.388 | 42.56 | 0.977 | 2-way ANOVA | TAM x Reporter | F (2, 6) = 0.2455 | P=0.7898 | No |
| 3-1C | 3D Long | 3 | 41.58 | 6.623 | 43.18 | 10.35 |  | TAM | F (2, 6) = 3.436 | P=0.1013 | No |
| 3-1C | 5D | 3 | 54.77 | 6.252 | 60.04 | 8.071 |  | Reporter | F (1, 6) = 1.517 | P=0.2642 | No |
|  |  |  |  |  |  |  |  | Subject | F (6, 6) = 2.179 | P=0.1829 | No |
|  | | | | | | | | | | | |
| EYFP and tdTomato DG Progenitor Density Correlation | | | | | | | | | | | |
| Figure | TAM | n | test | statistic | p value | Significant? |  |  |  |  |  |
| 3-1D | All | 9 | Pearson's Correlation | r(8) = 0.6308 | p = 0.0685 | No |  |  |  |  |  |
|  | | | | | | | | | | | |
| DG Percent of RGLs with True+ Signal | | | | | | | | | | | |
| Figure | TAM | n | RGLs Mean True + Percent | SE | test | Comparison | statistic | p value | Significant? |  |  |
| 4-1B | 3D Short | 3 | 14.71 | 5.241 | 2-way ANOVA | true signal x tam | F (2, 6) = 6.678 | P=0.0298 | Yes * |  |  |
| 4-1B | 3D Long | 3 | 15.85 | 5.037 |  | true signal | F (1, 6) = 4.012 | P=0.0920 | Yes * |  |  |
| 4-1B | 5D | 3 | 38.33 | 6.566 |  | tam | F (2, 6) = 1.235 | P=0.3554 | No |  |  |
|  |  |  |  |  |  | Subject | F (6, 6) = 0.02675 | P=0.9998 | No |  |  |
|  |  |  |  |  | Post-hoc | Comparisons | Adjusted p value | Significant? |  |  |  |
|  |  |  |  |  | Tukey's multiple comparisons | 3D short vs. 3D long | p = 0.988 | No |  |  |  |
|  |  |  |  |  |  | 3D short vs. 5D | p = 0.0239 | Yes * |  |  |  |
|  |  |  |  |  |  | 3D long vs. 5D | p = 0.0311 | Yes * |  |  |  |
|  | | | | | | | | | | | |
| DG Percent of RGLs with True- Signal | | | | | | | | | | | |
| Figure | TAM | n | RBLs Mean True - Percent | SE | test | Comparison | statistic | p value | Significant? |  |  |
| 4-1B | 3D Short | 3 | 42.49 | 5.636 | 2-way ANOVA | true signal x tam | F (2, 6) = 6.678 | P=0.0298 | Yes * |  |  |
| 4-1B | 3D Long | 3 | 44.69 | 5.241 |  | true signal | F (1, 6) = 4.012 | P=0.0920 | Yes * |  |  |
| 4-1B | 5D | 3 | 18.8 | 4.559 |  | tam | F (2, 6) = 1.235 | P=0.3554 | No |  |  |
|  |  |  |  |  |  | Subject | F (6, 6) = 0.02675 | P=0.9998 | No |  |  |
|  |  |  |  |  | Post-hoc | Comparisons | Adjusted p value | Significant? |  |  |  |
|  |  |  |  |  | Tukey's multiple comparisons | 3D short vs. 3D long | p = 0.956 | No |  |  |  |
|  |  |  |  |  |  | 3D short vs. 5D | p = 0.0235 | Yes * |  |  |  |
|  |  |  |  |  |  | 3D long vs. 5D | p = 0.014 | Yes * |  |  |  |
|  | | | | | | | | | | | |
| DG Percent of RGLss with True+/- Signal | | | | | | | | | | | |
| Figure | TAM | n | RGL Mean True +/- Percent | SE | test | statistic | p value | Significant? |  |  |  |
| 4-1C | 3D Short | 3 | 57.21 | 1.595 | One-way ANOVA | F (2, 6) = 1.235 | P=0.3554 | No |  |  |  |
| 4-1C | 3D Long | 3 | 60.54 | 1.464 |  | F (2, 6) = 1.235 | P=0.3554 | No |  |  |  |
| 4-1C | 5D | 3 | 57.13 | 2,117 |  | F (2, 6) = 1.235 | P=0.3554 | No |  |  |  |
|  | | | | | | | | | | | |
| DG Percent of Progenitors with True+ Signal | | | | | | | | | | | |
| Figure | TAM | n | Prog. Mean True + Percent | SE | test | Comparison | statistic | p value | Significant? |  |  |
| 4-1D | 3D Short | 3 | 13.45 | 2.997 | 2-way ANOVA | true signal x tam | F (2, 6) = 3.436 | P=0.1013 | No |  |  |
| 4-1D | 3D Long | 3 | 22.27 | 8.225 |  | true signal | F (1, 6) = 1.638 | P=0.2478 | No |  |  |
| 4-1D | 5D | 3 | 34.72 | 2.792 |  | tam | F (2, 6) = 1.927 | P=0.2258 | No |  |  |
|  |  |  |  |  |  | Subject | F (6, 6) = 0.08946 | P=0.9951 | No |  |  |
|  | | | | | | | | | | | |
| DG Percent of Progenitors with True- Signal | | | | | | | | | | | |
| Figure | TAM | n | Prog Mean True - Percent | SE | test | Comparison | statistic | p value | Significant? |  |  |
| 4-1D | 3D Short | 3 | 37.27 | 1.434 | 2-way ANOVA | true signal x tam | F (2, 6) = 3.436 | P=0.1013 | No |  |  |
| 4-1D | 3D Long | 3 | 37.51 | 8.63 |  | true signal | F (1, 6) = 1.638 | P=0.2478 | No |  |  |
| 4-1D | 5D | 3 | 19.91 | 5.977 |  | tam | F (2, 6) = 1.927 | P=0.2258 | No |  |  |
|  |  |  |  |  |  | Subject | F (6, 6) = 0.08946 | P=0.9951 | No |  |  |
|  | | | | | | | | | | | |
| DG Percent of RGLs with True+/- Signal | | | | | | | | | | | |
| Figure | TAM | n | Prog. Mean True +/- Percent | SE | test | statistic | p value | Significant? |  |  |  |
| 4-1E | 3D Short | 3 | 50.72 | 3.567 | One-way ANOVA | F (2, 6) = 1.927 | p = 0.2258 | No |  |  |  |
| 4-1E | 3D Long | 3 | 59.77 | 0.4557 |  | F (2, 6) = 1.927 | p = 0.2258 | No |  |  |  |
| 4-1E | 5D | 3 | 54.63 | 4.381 |  | F (2, 6) = 1.927 | p = 0.2258 | No |  |  |  |
|  | | | | | | | | | | | |
| EYFP and tdTomato SVZ Percent area Correlation | | | | | | | | | | |  |
| Figure | TAM | n | test | statistic | p value | Significant? |  |  |  |  |  |
| 4-2B | 5D | 10 | Pearson's Correlation | r(9) = 0.7809 | p = 0.0077 | Yes ** |  |  |  |  |  |
|  | | | | | | | | | | | |
| SVZ EYFP+ Percent Area Colocalization with tdTomato+ Area | | | | | | | | | | |  |
| Figure | TAM | n | Colocalized Area | Standard Error | test | statistic | p value | Significant? |  |  |  |
| 4-2C | 5D | 10 | 57.01 | 3.715 | One-Sample t-test w/ comparison to 100% | t(9)= 15.08 | p < 0.0001 | Yes **** |  |  |  |
|  | | | | | | | | | | | |
| SVZ tdTomato+ Percent Area Colocalization with EYFP+ Area | | | | | | | | | | | |
| Figure | TAM | n | Colocalized Area | Standard Error | test | statistic | p value | Significant? |  |  |  |
| 4-2D | 5D | 10 | 40.63 | 4.348 | One-Sample t-test w/ comparison to 100% | t(9)= 9.115 | p = 0.0001 | Yes **** |  |  |  |
