# Supplementary figures and images for: Poor Concordance of Floxed Sequence Recombination in Single Neural Stem Cells: Implications for Cell Autonomous Studies
Source: eNeuro. 2020 Mar 13;7(2):ENEURO.0470-19.2020. doi: 10.1523/ENEURO.0470-19.2020 (PMC7086402; doi:10.1523/ENEURO.0470-19.2020)

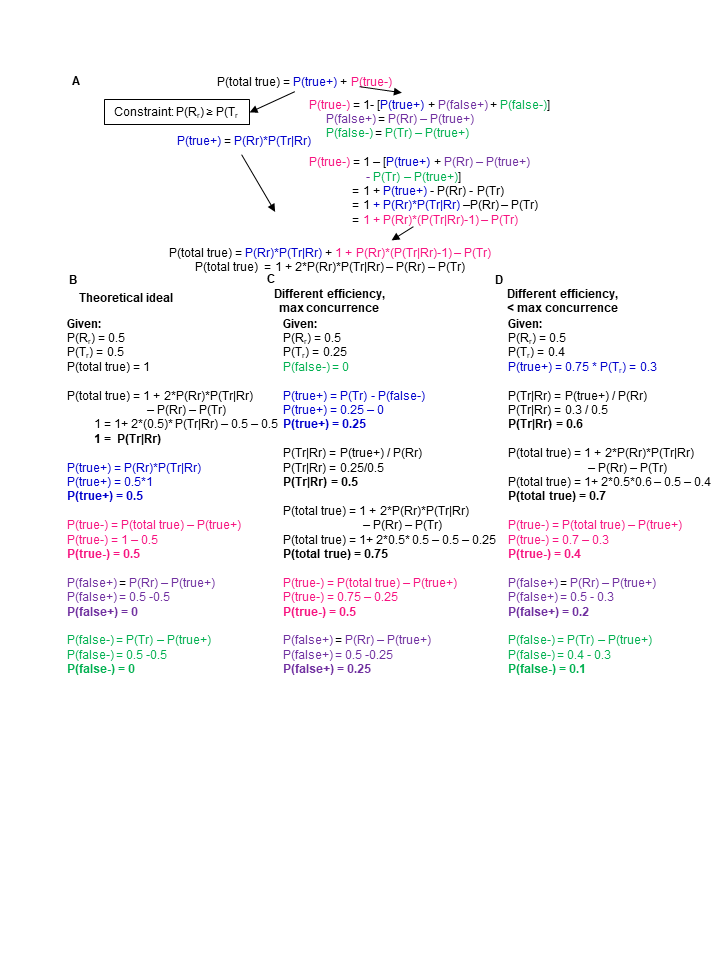

Supplement: Extended Data Figure 1-1 — Equation derivation for true and false signal probabilities. A, Assuming reporter recombination is greater than or equal to target gene recombination [P(Rr) ≥ P(Tr)], probability of total true signal is derived using standard conditional and unconditional probability formulas. B–D, Application of the equations in A to the scenarios described in Figure 1B are shown in full detail. Figure Contributions: Elizabeth Diana Kirby developed theoretical models. Download Figure 1-1, TIF file. [file enu-eN-TNC-0470-19-s02.tif]

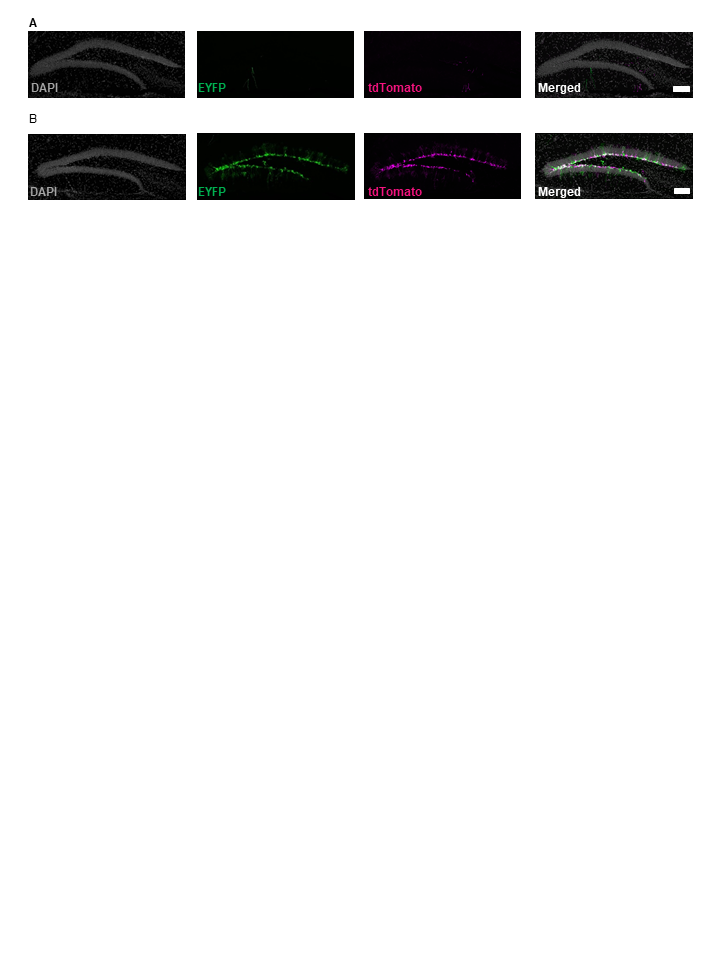

Supplement: Extended Data Figure 2-1 — Comparison of oil- and TAM-injected adult NestinCreERT2;Rosa(EYFP/tdTom) mice. A, Immunostaining in the adult DG shows that oil administration does not stimulate expression of either reporter gene. B, TAM administration induces robust recombination-dependent expression of both EYFP and tdTomato in the SGZ. Scale bars: 100 μm. Figure Contributions: Tyler Joseph Dause ran TAM experiments and tissue staining. Tyler Joseph Dause and Elizabeth Diana Kirby made figures. Download Figure 2-1, TIF file. [file enu-eN-TNC-0470-19-s03.tif]

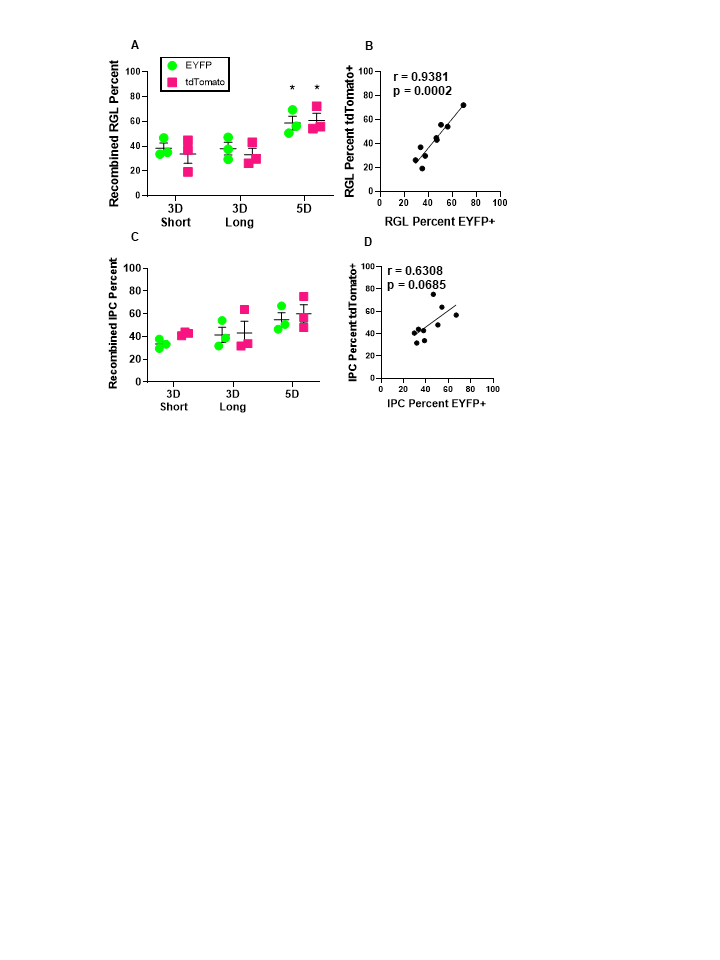

Supplement: Extended Data Figure 3-1 — Cell-specific fluorescent reporter recombination frequency and correlation. A, Percent of EYFP+ or tdTomato+/GFAP+ RGLs were compared in 3D short, 3D long, and 5D mice. B, Correlation of percent of GFAP+ RGLs that express EYFP and tdTomato in all mice. C, Percent of EYFP+ or tdTomato+ IPCs were compared in 3D short, 3D long, and 5D mice. D, Correlation of percent of Ki67+ IPCs that express EYFP and tdTomato in all mice; n = 3 mice per group. Data are shown as mean ± SEM; *p < 0.05, determined by two-way ANOVA (A, C) or Pearson’s correlation (B, D). Figure Contributions: Tyler Joseph Dause ran experiments and analyzed data. Tyler Joseph Dause and Elizabeth Diana Kirby made figures. Download Figure 3-1, TIF file. [file enu-eN-TNC-0470-19-s04.tif]

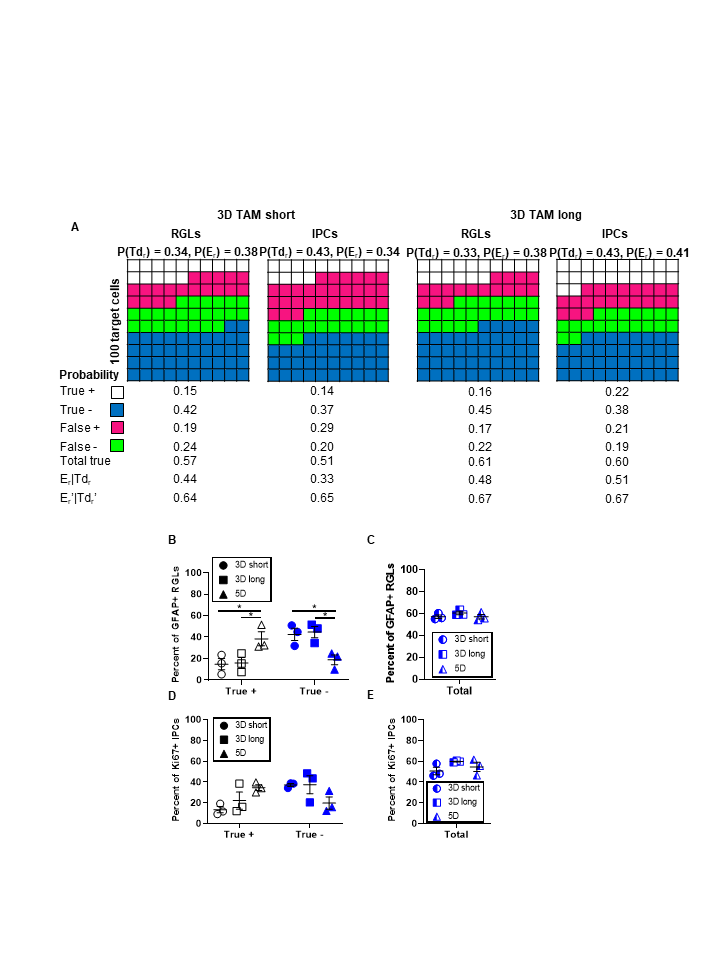

Supplement: Extended Data Figure 4-1 — Effects of cell type and TAM protocol on accuracy of using expression of one reporter to predict the other. A, The mean observed recombination frequencies from the 3D short (left) and 3D long (right) groups are represented here as reporter expression in 100 hypothetical target cells, either RGLs or IPCs. The probabilities of true and false signals are given as if tdTomato expression is being used to predict EYFP expression. B, The percent of GFAP+ RGL cells that show recombination in both reporter genes (true +) or neither (true –) is shown for three TAM groups. C, The total true signal (+ and –) in GFAP+ RGL cells for the three TAM groups is shown. D, The percent of Ki67+ IPCs that show recombination in both reporter genes (true +) or neither (true –) is shown for three TAM groups. E, The total true signal (+ and –) in Ki67+ IPCs for the three TAM groups is shown; n = 3 mice per group. Data shown are mean ± SEM; *p < 0.05, determined by two-way ANOVA. Figure Contributions: Tyler Joseph Dause ran experiments and analyzed data. Tyler Joseph Dause and Elizabeth Diana Kirby made figures. Download Figure 4-1, TIF file. [file enu-eN-TNC-0470-19-s05.tif]

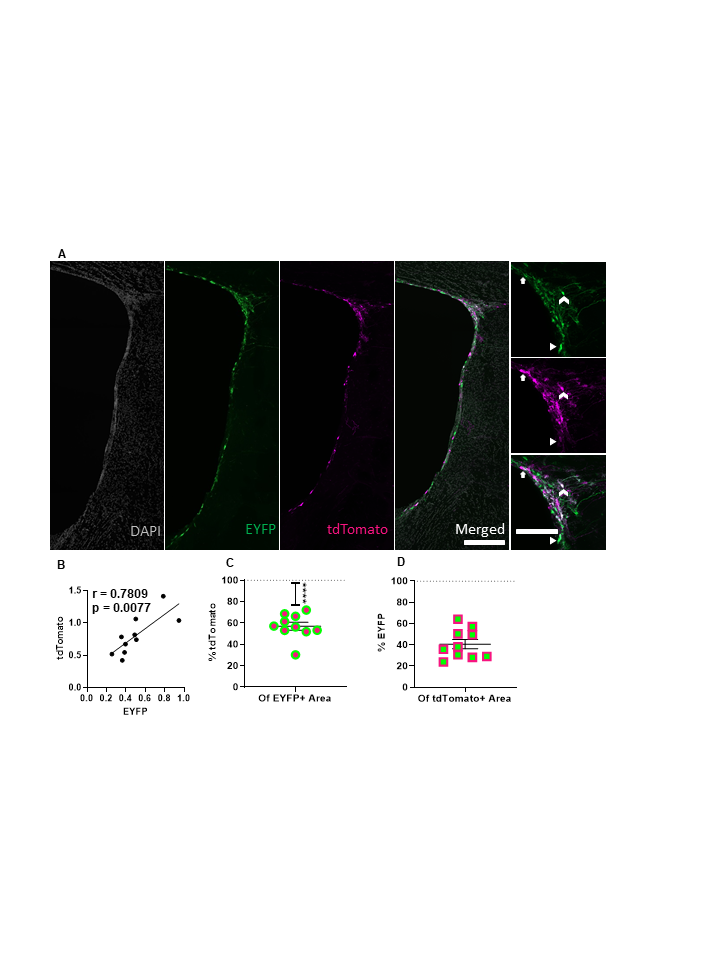

Supplement: Extended Data Figure 4-2 — Fluorescent reporter recombination and colocalization in SVZ NSPCs. A, Immunostaining of EYFP, tdTomato in SVZ NSPCs. Scale bars: 100 μm. Arrowhead = EYFP+/tdTomato– NSPC. Arrow = EYFP–/tdTomato+ NSPC. Chevron = EYFP+/tdTomato+ NSPC. B, Correlation of EYFP+ and tdTomato+ SVZ percent area in all mice. C, Comparison of EYFP+ colocalization in tdTomato+ area to theoretical 100% colocalization. D, Comparison of tdTomato+ colocalization in EYFP+ area to theoretical 100% colocalization; n = 9 mice. Data are shown as mean ± SEM; ****p < 0.0001 determined by Pearson’s correlation (B) or one-sample t test against a theoretical 100% (C, D). Figure Contributions: Tyler Joseph Dause ran experiments and analyzed data. Tyler Joseph Dause and Elizabeth Diana Kirby made figure. Download Figure 4-2, TIF file. [file enu-eN-TNC-0470-19-s06.tif]
